# Ionizing radiation effects on blood-derived extracellular vesicles: insights into miR-34a-5p-mediated cellular responses and biomarker potential

**DOI:** 10.1186/s12964-024-01845-x

**Published:** 2024-10-02

**Authors:** Chiara Huber, Omar Elsaeed, Pia Lahmer, Simone Moertl

**Affiliations:** Department of Effects and Risks of Ionizing & Non-Ionizing Radiation, Federal Office for Radiation Protection (BfS), Neuherberg, Germany

**Keywords:** MiR-34a-5p, Keratinocytes, Exosomes, Intercellular communication, PBMC, Bystander effect, Ionizing radiation, MiRNA, Radiation response, Signalling

## Abstract

Adverse effects of ionizing radiation on normal tissues limit the radiation dose in cancer treatment, thereby compromising treatment efficiency. Among the consistently affected non-cancer cells, peripheral blood mononuclear cells (PBMCs) exhibit high radiosensitivity and have the potential to induce systemic effects. PBMC-released extracellular vesicles (EVs), contribute to the communication of such systemic effects. This study aimed to investigate the effects of ionizing radiation on EVs as part of the systemic response of PBMCs in terms of microRNA cargo and biological functions.

Therefore, whole blood samples from healthy donors were irradiated ex-vivo (0 Gy, 1 Gy, 2 Gy, 4 Gy) and EVs from PBMCs were isolated after 96 h by PEG precipitation or ultracentrifugation. Candidate microRNAs were examined in PBMC-derived EVs from individual donors. The uptake of membrane-stained fluorescent EVs by different recipient cells was quantified by fluorescence-activated cell sorting analysis. The biological effects of increased miR-34a-5p and of total EVs on recipient cells were assessed.

Irradiation of PBMCs induced a dose-dependent upregulation of miR-34a-5p within EVs and PBMCs. However, interindividual differences between donors were noticed in the extent of upregulation, and small EVs displayed more pronounced changes in microRNA levels in comparison to large EVs. Irradiation in presence of the small molecule inhibitor KU-60019 demonstrated that this upregulation is dependent on ATM (Ataxia telangiectasia mutated) activation. Moreover, fibroblasts and keratinocytes were identified as preferred EV recipients. Increased miR-34a-5p levels led to a significant reduction in viability and induction of senescence in keratinocytes but not in fibroblasts, indicating a cell type-specific response.

In conclusion, this study further elucidated the complex cellular response of normal tissue after radiation exposure. It confirmed radiation-induced modifications of microRNA expression levels in EVs from PBMCs and identified a robust upregulation of miR-34a-5p in the small EV subfraction, suggesting this microRNA as a potential novel candidate for the development of biomarkers for radiation exposure. Moreover, the different uptake efficiencies observed among specific cell types suggested that EVs induce cell type-specific responses in the intercellular communication of systemic radiation effects.

**Supplementary Information:**

The online version contains supplementary material available at 10.1186/s12964-024-01845-x.

## Introduction

Radiation therapy is a frequently used cancer treatment, with nearly 50% of all cancer treatments involving radiotherapy [1]. However, radiotherapy not only affects cancer cells but also surrounding normal tissue, resulting in adverse effects that impair treatment effectiveness and patient’s quality of life. Understanding the underlying mechanisms of normal tissue effects can help to improve treatment outcomes, including the identification of patients at risk and the development of strategies to mitigate normal tissue effects.

Among the normal tissues affected by radiotherapy, immune cells play a particularly important role as they are present in the tumor, its microenvironment, and the nearby blood supply system. Immune cells are generally considered radiation-sensitive and are often used as surrogate tissues in radiation biology, for example in the discovery of biomarkers for radiation sensitivity and biodosimetry applications [2, 3]. Traditionally, radiation effects on immune cells were described as immune suppressive. Nevertheless, more recent insights revealed complex additional roles for irradiated immune cells, including radiation-induced antitumor immunity [4].

On the cellular level, radiation triggers various responses, including cell cycle arrest, DNA repair, or cell death in irradiated cells, mainly through Ataxia Telangiectasia Mutated (ATM)-mediated response [5]. Importantly, these effects are not restricted to (directly) targeted cells but can also occur indirectly in neighboring non-irradiated cells, known as the radiation-induced bystander effect (RIBE) [6]. Indirect radiation effects can lead to similar biochemical and phenotypic changes in cells or tissues as observed in directly irradiated cells [7]. However, beneficial RIBEs have also been observed. For example, the secretome of irradiated peripheral blood mononuclear cells (PBMCs) can attenuate cardiovascular diseases by reducing inflammation and promoting angiogenesis and wound healing. These properties are caused by changes in the PBMC secretome, which include released proteins like cytokines and chemokines [8]. Furthermore, extracellular vesicles (EVs) released by immune cells have recently gained increasing attention as mediators of these radiation-induced systemic effects [9–11].

EVs are small membrane-surrounded vesicles that play an important role in intercellular communication by carrying various molecules such as proteins, nucleic acids, metabolites, and lipids [12, 13]. Depending on their cellular origin and size, EVs can be grouped into exosomes, microvesicles, and apoptotic bodies. Once released, they can interact with surface receptors or transfer the encapsulated cargo to recipient cells [14], thereby participating in cell–cell communication with adjacent or distant cells. EVs are an essential part of the cellular stress response, and their cargo and function change in response to stress [15, 16], including radiation exposure [17–19]. EVs released by irradiated cells can affect the behavior of recipient cells for example in terms of proliferation, survival, senescence, and migration [9, 18, 20, 21]. A well-known EV cargo that has been reported to influence the behavior of recipient cells are microRNAs (miRNAs), which are 19 to 25 nucleotides long non-coding RNAs that regulate gene expression at the post-transcriptional level [22, 23]. Circulating EVs with miRNA cargo have gained interest as potential markers for physiological stress and pathological conditions.

A recent pilot study already described radiation-induced changes in the protein and miRNA cargo of EVs released from immune cells by label-free mass spectrometry and RNA sequencing in a limited number of donors [10]. The objective of this study was to examine the effects of radiation on EVs across age and gender groups, as well as within various EV subfractions. Additionally, potential EV target cells and EV-induced biological effects in recipient cells were addressed. Our results showed a robust radiation-induced upregulation of miRNA miR-34a-5p in the tested healthy donors, thus recommending miR-34a-5p as a potential new exposure biomarker candidate. Moreover, fibroblasts and keratinocytes were identified as preferred recipient cells with miR-34a-5p effects leading to decreased viability and increased senescence.

## Materials and methods

### Cell culture

PBMCs were kept in RPMI 1640 medium with stable glutamine, 2.0 g/L NaHCO_3_ (PAN Biotech, Germany), and 13% heat-inactivated fetal bovine serum (FBS Premium, PAN-Biotech, Germany). For counting, cells were mixed with an equal volume of Tuerk's solution (Thermo Fisher Scientific, USA) and counted using a Neubauer hemocytometer.

For experiments involving vesicle isolation from the cell culture supernatant, vesicle-free FBS was used. Therefore, FBS was ultrafiltered using an ultrafiltration unit (Amicon Ultra-15, Merck Millipore, USA) with a molecular weight cut-off of 100 kDa.

Human skin fibroblasts 1BR3 (ECACC 90011801, Sigma-Aldrich, USA) were grown in Eagle's minimum essential medium (EMEM, Sigma-Aldrich, USA) supplemented with 15% FBS, 2 mM L-Glutamine (Gibco, Thermo Fisher Scientific, USA) and 1% Non Essential Amino Acids (Gibco, Thermo Fisher Scientific, USA). MRC-5 fetal lung fibroblasts were grown in Dulbeccos Modified Eagle Medium (DMEM)/F-12 (1:1) (Gibco, Thermo Fisher Scientific, USA) with 10% FBS. Immortalized oral keratinocytes OKF6 TERT-1 were grown in keratinocyte serum-free medium supplemented with 25 μg/ml bovine pituitary extract and 0.2 ng/ml epidermal growth factor (Gibco, Thermo Fisher Scientific, USA). Human coronary fibroblasts (HCF) were grown in Fibroblast Growth Medium 3 (Promo Cell, Germany). Human umbilical vein endothelial cells (HUVEC) were grown in endothelial cell growth medium 2 (PromoCell, Germany). Human coronary artery endothelial cells (HCAECs) were grown in MesoEndo Cell Growth Medium (Cell Applications, USA). The oral squamous cell carcinoma cell lines Cal33 and BHY were grown in DMEM high glucose (PAN-Biotech, Germany) supplemented with 10% FBS and 2 mM L-Glutamine. All cells were grown at 37 °C and 95% humidity with 5% CO_2_ for PBMCs, 1BR3, MRC-5, OKF6 TERT-1, HCF, HUVEC and HCAECs or 10% CO_2_ for BHY and Cal33. All cell lines (except 1BR3) were provided by the Institute of Radiation Biology (ISB) at the Helmholtz Center Munich (HMGU) and tested regularly for mycoplasma contamination.

For passaging adherent cell lines, cells were washed once with Dulbecco’s Phosphate Buffer Saline (DPBS, Biowest, France), detached at 37 °C using TrypLE Express enzymes (Thermo Fisher Scientific, USA), and centrifuged at 200 g to remove the enzymes before they were resuspended in growth medium.

### Irradiation

All samples were irradiated in flasks, plates, or blood tubes in horizontal positions using the X-ray cabinet RS225 (Xstrahl Limited, UK). Irradiation was performed with a tube voltage of 195 kV, 10 mA current, and a 0.5 mm copper filter plus an additional 2.09 mm flattening aluminum filter at room temperature (RT). The distance of the samples from the source was set to 500 mm to provide a dose rate of 0.52 Gy/min.

### Blood sample collection and PBMC isolation

Whole blood from 12 healthy donors (5 male, 7 female) aged between 20 and 63 was collected in 7.5 mL sterile Lithium-Heparin S-monovettes (#01.1604.001, Sarstedt, Germany). Blood samples taken on different days from randomly selected individuals from these 12 donors were defined as biological replicates. All blood samples were directly irradiated ex vivo in monovettes immediately before PBMCs were isolated.

PBMCs were isolated immediately following irradiation by density gradient centrifugation using Histopaque-1077 (Sigma-Aldrich, USA). 15 mL of Histopaque-1077 were added to a 50 mL Leucosep tube (Greiner Bio-one, Austria) and centrifuged for 1 min at 1000 g. Whole blood was diluted in DPBS (1:1) and layered onto the porous barrier before centrifugation without a brake at 400 g for 30 min at 20 °C. Following centrifugation, the plasma layer was discarded, and the buffy coat, which contains the PBMCs, was collected with a pasteur pipette and transferred to a tube containing 10 mL of RPMI 1640 medium. To minimize residual platelet contamination, PBMCs were washed in RPMI 1640 and centrifuged twice at 250 g for 10 min. For vesicle analysis, cells were adjusted to a concentration of 10^6^ cells/mL in RPMI 1640 medium supplemented with 13% vesicle-free FBS (see section Cell culture) and incubated for 24–96 h. To harvest PBMCs, cells were centrifuged at 300 g for 10 min and washed twice with DPBS before the pellet was stored at -80 °C.

### Ataxia Telangiectasia Mutated (ATM) Inhibitor treatment

The ATM inhibitor KU-60019 (Sigma-Aldrich, USA) was prepared in a concentration of 30 mM in dimethyl sulfoxide (DMSO, PAN-Biotech, Germany) and stored in aliquots at -20 °C until use. Before treatment, PBMCs were diluted to 10^6^ cells/mL in vesicle-free medium. Either 3 µM KU-60019 or 0.01% DMSO as a solvent control was added to the PBMCs. The cells were incubated at 37 °C for 1 h and subsequently exposed to 2 Gy or sham irradiation.

### Isolation of EVs

To isolate EVs, conditioned cell culture medium was centrifuged at 300 g for 10 min at RT to separate cells from the supernatant. Cell debris was removed at 3000 g for 10 min. Total EVs (tEVs) were isolated directly from the supernatant through PEG precipitation or ultracentrifugation (UC). Alternatively, the tEVs were further separated into large EVs (lEVs) and small EVs (sEVs) through centrifugation at 10,000 g for 30 min at 4°C. The pellet containing lEVs was washed once with sterile-filtered (0.1 µm) DPBS, centrifuged again at 10,000 g for 30 min, and stored at -80 °C for further analysis. sEVs were isolated from the supernatant by PEG precipitation or UC.

### PEG Precipitation

The cell culture supernatant was concentrated using an ultrafiltration unit with a molecular weight cut-off of 100 kDa (Vivaspin2, Sartorius, Germany). To precipitate tEVs, a 40% w/v stock solution of polyethylene glycol 6000 (PEG, Sigma-Aldrich) dissolved in deionized water (30 °C) and sterile filtered (0.2 μm), was mixed with the supernatant to achieve a final concentration of 8% PEG. Tubes were incubated for 1 h at 4 °C and centrifuged for 30 min at 1500 g. The supernatant was aspirated and the tubes were centrifuged for another 5 min at 1500 g. The remaining supernatant was then completely removed with a 10 µL pipette tip, and the pellet was either stored or resuspended in 30–100 µL of sterile-filtered (0.1 µM) DPBS. The suspension was mixed by scratching over a rack and stored at -80 °C for further analysis.

### Ultracentrifugation (UC)

For EV isolation by UC, cell culture supernatants depleted of cells, debris, and optionally lEVs, were transferred into polycarbonate tubes (#5082 or #4494, Seton Scientific Petaluma, USA) and centrifuged at 100,000 g for 90 min at 4 °C in a T-1250 fixed-angle rotor (k-factor 68.7) and Sorvall WX ultracentrifuge (Thermo Fisher Scientific, USA). The pellet was washed in 1 mL sterile-filtered (0.1 µm) DPBS and transferred to a 1.5 mL polypropylene tube (#357,448, Beckman Coulter, USA). After another round of centrifugation for 90 min at 100,000 g using 1.5 mL microfuge tube adapters (#11,058, Beranek Laborgeräte, Germany), the pellet was stored or resuspended in 30–100 µL sterile-filtered DPBS at – 80 °C.

### Nanoparticle tracking analysis

The Zetaview twin nanoparticle tracking analyzer (NTA) from Particle Metrics (Germany) was used to measure particle concentration, size distribution, and presence of the vesicle-marker CD63 using the green (520/550 nm) laser/filter combination. All samples were diluted in DPBS to a final volume of 1 mL and around 250 particles per frame. The following settings were used for scattered light: Camera sensitivity: 85, Shutter: 100.

In order to stain membrane-containing particles, 1 µL prediluted (1:500) CellMask Orange (CMO, Invitrogen, Thermo Fisher Scientific, USA) was added to the sample in a total volume of 10 µL and incubated for 5 min at RT. The sample was then further diluted to get a final concentration of 10 ng/µL CMO and approximately 250 particles (Scatter) per frame. Fluorescence was analyzed at a sensitivity of 93 and a Shutter of 100.

For CD63 antigen staining, samples were adjusted to a concentration of approximately 1000 particles per frame in Scatter mode. Samples were mixed with 1 µL prediluted (1:10) CD63 antibody (Miltenyi Biotec, Germany) and incubated for 30 min at RT. Samples were further diluted to 1 mL (CD63 dilution 1:10,000). Fluorescence was analyzed at a sensitivity of 93. Videos were analyzed by the ZetaView Software version 8.0.16 with the following parameters based on the manufacturer’s pre-set parameters: particle size: 5–1000 nm, minimum brightness: 30 (Scatter/CMO) or 25 (CD63), minimal area of particles: 30, minimal tracking length: 15 frames. All samples were individually thawed right before analysis and kept on ice. To analyze EV release, the concentrations of unstained (scatter) and CMO-stained particles were measured. The relative particle changes have been further normalized to the number of viable cells per time point by dividing the particle concentrations with the cell counts determined at the time of EV harvest. All values were normalized to the unirradiated samples at 48 h.

### RNA isolation

Total RNA isolation was performed using the mRNeasy Kit (Qiagen, Germany) according to the manufacturer’s protocol. MiRNA of cells and EVs were extracted using the miRNeasy micro Kit (Qiagen, Germany) as stated in the manufacturer’s protocol.

### Reverse transcription of mRNA/miRNA and quantitative real-time polymerase chain reaction (qPCR)

Total RNA was transcribed to cDNA with the GoScript Reverse Transcription System (Promega, USA) and miRNA was transcribed using the miCURY LNA RT Kit (Qiagen, Germany) according to the manufacturer’s instructions. Quantitative PCR was performed on a CFX96 touch real-time PCR detection system (Bio-Rad, USA) using the iTaq SYBR Green Mastermix (Bio-Rad Laboratories, USA) and a calculated amount of 1–10 ng cDNA per reaction. MiRNA primers from the miRCURY LNA miRNA PCR Assays were purchased from Qiagen, whereas mRNA primers were synthesized by Eurofins genomics or purchased from Qiagen’s QuantiTect Primer Assays (see Supplementary Table 1).

### Western blotting

For western blot analysis, cells or EVs were lysed in RIPA lysis buffer containing 1% Halt Protease and Phosphatase Inhibitor Cocktail (Thermo Fisher Scientific, USA) and 5 mM EDTA for 15 min on ice. The cell and vesicle lysates were centrifuged for 15 min at 14,000 g. The protein concentration was determined by the DC protein assay (Bio-Rad Laboratories, USA). Proteins were denatured by heating with 4 × Laemmli Sample buffer (Bio-Rad Laboratories, USA) supplemented with 2 mM dithiothreitol (DTT) for 10 min at 95 °C.

For protein separation by sodium dodecyl sulfate–polyacrylamide gel electrophoresis (SDS-PAGE), 20–30 µg of sample proteins, were loaded onto 4–20% Criterion TGX Stain-Free Precast Gels (Bio-Rad Laboratories, USA), allowing for total protein normalization. The gels were run in TGS buffer (250 mM Tris, 1.92 M Glycine, 0.1% SDS, pH 8.3) at 50 V for 15 min and 200 V for 40 min.

Proteins were transferred onto a polyvinylidene difluoride membrane (Immun-Blot Low fluorescence PVDF Membrane, Bio-Rad Laboratories, USA) using the Trans-Blot Turbo Transfer System (Bio-Rad Laboratories, USA). The membranes were blocked with Intercept blocking buffer (LI-COR, USA) for 1 h at RT. Protein detection with primary antibodies was performed overnight at RT using the antibodies listed in Supplementary Table 2. For visualization, the fluorescent secondary antibody Starbright Blue 520/700 Goat-anti mouse/rabbit (1:10,000, Bio-Rad Laboratories, USA) and the ChemiDoc Imaging System (Bio-Rad Laboratories, USA) were used.

### EV–recipient cell interactions

To study the interactions between EVs and recipient cells, tEVs of 2 Gy or sham-irradiated PBMCs isolated after 96 h by ultrafiltration and PEG precipitation or UC were counted by NTA and diluted to a concentration of 1*10^11^ Particles/mL in a dilution of PKH26 in Diluent C (1:500, MINI26-1KT, Sigma-Aldrich, USA) to stain the EV membrane. As a negative control, equal volumes of PBS instead of EVs were mixed with PKH26 in Diluent C. Excessive dye was removed by using Exosome Spin Columns (Thermo Fisher Scientific, USA) according to the manufacturer’s protocol. Subsequently, stained EVs and PBS were further diluted 1:5 with DPBS.

For quantifying the EV uptake by FACs analysis, recipient cell lines (PBMCs, HCAEC, HUVEC, HCF, MRC-5, 1BR3, OKF6, BHY, Cal33) were seeded at a density of 100,000 cells per well in a 12-well plate in 2 mL medium (≙ 10^4^ EVs/cell). The next day, the medium was changed and 50 µL of stained EVs or control were added to the medium. After 24 h, cells were trypsinized, washed with DPBS, and fixed with 2% Paraformaldehyde (PFA, Sigma-Aldrich, USA) in DPBS for 15 min at RT. After washing with DPBS, cells were resuspended in 100 µL autoMACS FACS buffer, and the PKH26 signal was measured on a MACSQuant Analyzer 10 (Miltenyi Biotec, Germany) with an excitation of 488 nm in B2 channel (585/40 nm).

For visualizing EV uptake using fluorescence microscopy images, cells were seeded in a density of 10,000 cells in 100 µL medium per well of a 96-well plate. 5 µL of stained EVs were added (≙ 10^4^ EVs/cell), and cells were washed and fixed on the plate after 24 h. For high-resolution images of EV uptake by OKF6 cells, 10,000 cells of OKF6 in 100 µL were seeded in 8-well removable silicon chambers (Ibidi, Germany) attached to a polymer coverslip with ibiTreat tissue culture-treated surface (Ibidi, Germany). 10 µL of stained EVs were added to the cells and incubated for 24 h. Silicon chambers were removed before cells were washed in DPBS and fixed with 4% PFA for 10 min at RT. Ibidi slides were washed three times with DPBS and permeabilized by incubation with 0.1% Triton X-100 in DPBS for 10 min. After washing three times with DPBS, coverslips were incubated in a blocking solution with PBS containing 1% BSA, 300 mM glycine, and 0.1% Tween-20 for 30 min at RT. 50 µL of antibody Lamin A (1:100, ab26300, Abcam, UK) diluted in the blocking solution was added dropwise to the cells before they were covered and incubated for 1 h at RT. After another round of DPBS washing, cells were incubated with the secondary antibody Anti-rabbit IgG Alexa Fluor 488 (1:500, Cell Signaling Technology, USA) for 1 h at RT and mounted with 1 drop of VECTASHIELD Antifade Mounting Medium with DAPI (Vector Laboratories, USA) to stain the nuclei. The images were taken using a Zeiss AxioImager.Z2 microscope (Zeiss, Germany) coupled to an AxioCam 503 mono microscope camera together with the ZEN 3.5 Pro software. Nuclei were visualized in the DAPI channel, stained EVs were detected in the AF-555 channel, and the Lamin A signal was visualized using the AF-488 channel. For comparing the signal between different cell types, images were obtained with an EC Plan-Neofluar 20x/0.5 M27 objective. For a high-resolution z-stack image of OKF6, images were obtained with an EC Plan-Neofluar 100x/1.30 Oil Pol M27 objective in combination with Immersol 518 F (Zeiss, Germany).

### MicroRNA mimic transfection

For testing the effect of miR-34a-5p on viability, 3000 HCAEC and 1BR3, or 6000 OKF6 cells, respectively, were seeded in 100 µL medium per well in 96-well plates. For investigating the effect of miR-34a-5p on senescence and apoptosis, 100,000 or 200,000 cells were seeded in a 6-well plate containing 2.5 mL medium. The next day, the culture medium was replaced, and cells were transfected with 10 nM hsa-miR-34a mirVana miRNA mimic (#4,464,066, Thermo Fisher Scientific, USA) or 10 nM of a random sequence miRNA mimic negative control (mirVana miRNA mimic Negative Control #1, #4,464,058, Thermo Fisher Scientific, USA) using lipofectamine RNAiMAX (Thermo Fisher Scientific, USA), according to the manufacturer’s protocol.

### Biological effect of EVs on recipient cells

To assess the effect of irradiation on the biological function of PBMC-derived EVs, tEVs from the supernatant of 2 Gy and sham-irradiated PBMCs (10^6^ cells/mL) from four different donors were isolated by PEG precipitation after 96 h. The tEV pellet was resuspended in an equivalent volume of fresh OKF6 growth medium. 45,000 OKF6 cells were seeded in a 12-well plate in 1 mL of medium. The next day, OKF6 cells were washed with DPBS and the medium was replaced with 1 mL of tEVs isolated from 10^6^ PBMCs. OKF6 were irradiated 1 h after the addition of the EVs and harvested 72 h after irradiation to measure miR-34a-5p expression and effects on senescence.

### Cell viability assay

Cell viability was determined based on the average metabolic activity. The water-soluble tetrazolium (WST) assay was performed using WST-8 reagent (Cell Counting Kit-8, Sigma-Aldrich, USA), which produces an orange formazan product upon reduction by dehydrogenases.

Cells were seeded in 96-well plates with 100 µL of culture medium, followed by transfection and irradiation the day after seeding. As a control, untreated cells were used, and medium alone served as a blank. 72 h after treatment, 10 µL of the CCK-8 reagent was added to each well, and the samples were incubated for 3 h. The absorbance of the formazan product at 450 nm was subsequently quantified on a Tecan infinite plate reader (Tecan Group AG, Switzerland).

### Senescence assay

To measure senescence, β-galactosidase activity was evaluated in HCAEC, OKF6, and 1BR3 cell lines 72 h after treatment. For this purpose, the CellEvent Senescence Green Flow Cytometry Assay Kit (Invitrogen, Thermo Fisher Scientific, USA) was used to perform the assay according to the manufacturer's protocol. After harvesting and fixing the cells with 2% PFA, cells were stained for 1.5 h at 37 °C in a 1:500 working solution and analyzed using a 488 nm laser and the 525/50 nm filter (B1 channel) on the flow cytometer.

### Annexin V/PI apoptosis assay

The percentage of dead, early, and late apoptotic cells was determined using the Annexin-V-FITC Kit from Miltenyi according to the manufacturer’s protocol and analyzed on the flow cytometer. Annexin-positive and PI-negative cells were considered as early apoptotic cells, while annexin-positive and PI-positive cells were considered as late apoptotic cells.

### Data analysis

Western Blot band intensities were quantified using Image Lab version 6.1 (Bio-Rad Laboratories, USA) and normalized to the total protein load in each lane determined by the stain-free method. All flow cytometry data were analyzed on the MACSQuantify Software version 2.13.3. For qPCR analysis, ΔCt values were calculated as Ct_target_—Ct_housekeeper_, ΔΔCt values as ΔCt_treatment_- ΔCt_control_ and fold changes as 2^− ΔΔCt^. For statistical analysis, we employed various tests based on the specific comparisons required. To compare fold changes against a designated value, we utilized one-sample t-tests. For the comparison of two means, we applied the Student's t-test. When comparing two or more variables, we used a two-way ANOVA followed by the Benjamini–Hochberg post hoc test to control for false discovery rates. To compare multiple means against a single control mean, we conducted Dunnett's multiple comparison test. Data visualization and data analysis were performed using GraphPad Prism 9.5.0 software. *p ≤ 0.05; **p ≤ 0.01; ***p ≤ 0.001; ****p ≤ 0.0001.

## Results

### Isolation and characterization of total EVs isolated by PEG precipitation and UC

**Fig. 1:**
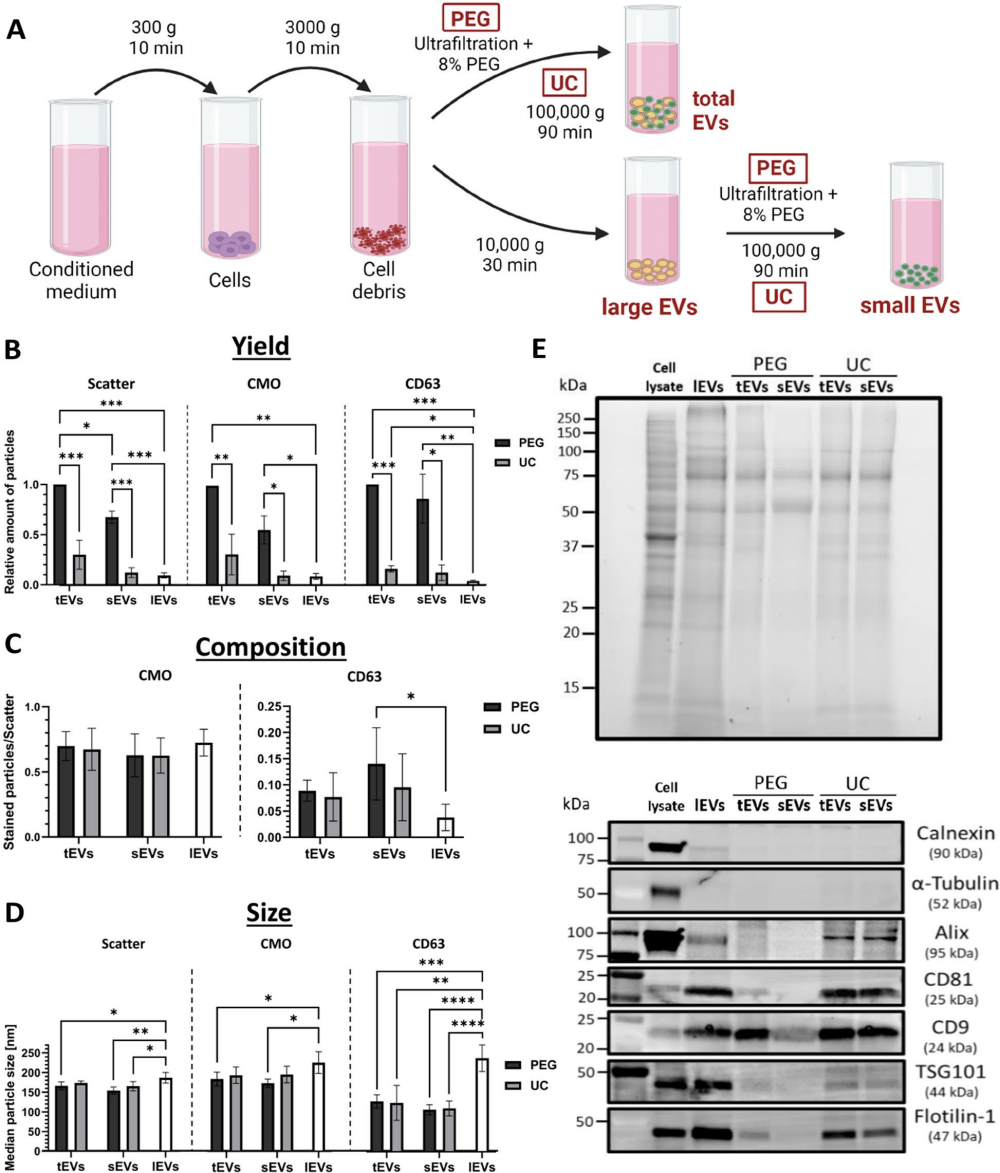
Characterization of EVs isolated by PEG precipitation or UC. **A** To isolate EVs, the conditioned cell culture medium was centrifuged at 300 g to pellet cells (PBMCs) and the supernatant was centrifuged again at 3000 g to remove cell debris and apoptotic bodies. TEVs were either directly isolated from the supernatant or another centrifugation step at 10,000 g was implemented to separate lEVs (pellet) from sEVs (supernatant) isolated by precipitation or UC. Created with Biorender.com **B** Comparison of tEVs, lEVs, and sEVs isolated by PEG precipitation or UC regarding the yield, **C** relative amount of CellMask Orange membrane (CMO) and CD63-stained particles, and **D** average particle size. Mean values ± SD of at least three biological replicates are shown. For statistical analysis, 2-way ANOVA and multiple t-tests with Benjamini–Hochberg correction were performed. **E** Total protein load after SDS-PAGE (top) and Western Blot (bottom) analysis of tEVs, lEVs, and sEVs fractions isolated by PEG-precipitation or UC from PBMC supernatants after 96 h. The positive EV markers, Programmed cell death 6-interacting protein (Alix), CD81, CD9, Tumor susceptibility gene 101 (TSG101), Flotillin-1 and non-EV markers, Calnexin (ER) and α-Tubulin, were used for immunoblotting. The all blue protein standard (Bio-Rad) was used for molecular weight estimation of the proteins

To investigate the effects of EVs in the radiation response of blood cells, EVs were isolated as shown in Fig. 1A. As the yield, purity, and functionality of EVs have been shown to be dependent on the isolation method [24, 25], EVs isolated by two different methods, either ultrafiltration combined with PEG precipitation or UC, were compared. To identify the vesicle subtype with the highest miRNA alterations for potential use as biomarkers for radiation exposure, both small extracellular vesicles (sEVs) and large extracellular vesicles (lEVs) were examined in addition to total extracellular vesicles (tEVs).

NTA results showed that a higher amount of tEVs and sEVs was isolated by PEG precipitation than by UC (Fig. 1B). In addition, the proportion of lEVs was relatively low compared to tEVs. These results were confirmed for membrane-positive (CMO) and CD63-positive particles. Concerning the composition of EVs, the proportion of membrane-stained particles among all particles detected in scatter mode remained the same for tEVs, lEVs, and sEVs, but the proportion of CD63-positive particles was higher in sEVs compared to lEVs (Fig. 1C).

The average median sizes of tEVs were similar for both isolation methods (Scatter: PEG 166 nm, UC 174 nm; CMO: PEG 186 nm, UC 196 nm; CD63: PEG 127 nm, UC 123 nm). As expected, sEVs were significantly smaller than lEVs with a median size of 154 nm (PEG) and 165 nm (UC), while lEVs had a median size of 186 nm (Fig. 1D). With a size around 110–130 nm, CD63-positive particles of tEVs and sEVs were smaller than lEVs and also smaller compared to CMO-stained or unstained particles. For all three EV subfractions, no EVs larger than 500 nm could be detected by NTA (Supplementary Fig. 1). The comparison between the histograms of the particle sizes of sEVs and lEVs showed an overall shift towards larger particles in the lEV fractions.

Additionally, several other vesicle markers could be detected by western blot analysis, indicating the successful isolation of EVs. CD81 and CD9 were enriched in the EV samples compared to the cell lysates (Fig. 1E). The absence of α-Tubulin and calnexin in the vesicle lysates indicates the absence of major impurities by cytoplasmic or endoplasmic reticulum (ER)-associated proteins.

### Irradiation increases miR-34a-5p in EVs of PBMCs

To investigate the effect of ionizing radiation on the miRNA cargo of EVs released by PBMCs, the expression of five radiation-responsive miRNAs was investigated. These miRNAs were selected based on their reproducible detectability, endogenous expression, and the extent of their radiation-induced expression change in a previous study [10]. MiRNA expression levels in tEVs as well as in the corresponding cells were quantified 96 h after irradiation with 1, 2, and 4 Gy or sham irradiation by quantitative real-time PCR. The miRNA cargo of EVs isolated by PEG precipitation or UC was compared for samples irradiated with a dose of 2 Gy. We focused on the 2 Gy dose as it is particularly significant, corresponding to the typical amount administered in a single radiotherapy session and marking a critical threshold in emergency radiation protection, beyond which medical treatment is necessary.

**Fig. 2:**
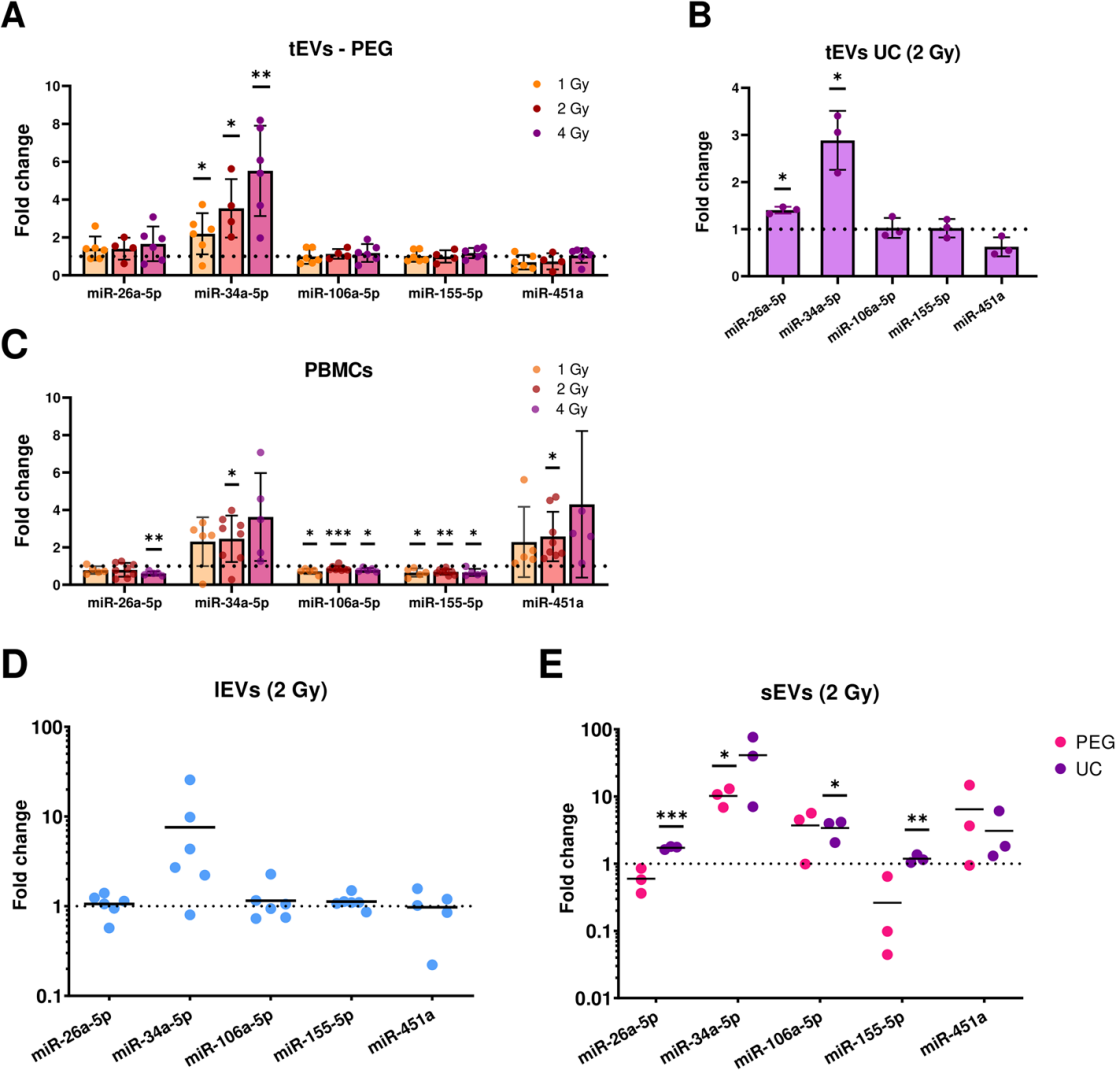
MiRNA expression in EVs and PBMCs. Fold changes of miRNA in tEVs isolated by **A**) PEG precipitation or **B**) UC and **C**) in the corresponding PBMCs 96 h after irradiation of whole blood with 1, 2, and 4 Gy. miRNA expression in **D**) lEVs and **E**) sEVs of PBMCs isolated by PEG precipitation or UC 96 h after irradiation of whole blood. Individual and mean values ± SD of at least four (tEVs), six (lEVs), or three (sEVs) biological replicates are shown. For statistical analysis, one sample t-tests were performed

Among the tested miRNAs, miR-34a-5p was significantly higher in tEVs in a dose-dependent manner, whereas the other miRNA levels were hardly affected by ionizing radiation (Fig. 2A, B). MiRNA levels in tEVs isolated by PEG precipitation (Fig. 2A) or UC (Fig. 2B) showed comparable results. In the corresponding irradiated PBMCs, miR-34a-5p was significantly upregulated (Fig. 2C) but no significant correlation between cellular and vesicle-associated miR-34a-5p levels could be observed (Supplementary Fig. 2). The observed changes in expression levels of miR-26a-5p, miR-106a-5p, miR-155-5p and miR-451a in PBMCs were not displayed in the tEVs (Fig. 2A, C).

To investigate whether distinct EV subpopulations differ in their miRNA cargo and to identify the optimal vesicle subpopulation for miRNAs as potential biomarkers of radiation exposure, we examined small and large EVs, in addition to total EVs (Fig. 2D and E).

Results showed variable, non-significantly increased miR-34a-5p levels in lEVs (Fig. 2D). Similar to the tEV miRNA content, no other tested miRNA was found radiation-responsive. sEVs isolated by either PEG precipitation or UC showed a 10–40 fold upregulation of miR-34a-5p levels, which is considerably higher than in the tEV fraction (Fig. 2A, E). Interestingly, expression levels of miR-26a-5p, miR-106a-5p, and miR-155-5p also appeared to be radiation-regulated in sEVs isolated by UC (Fig. 2E).

Further analysis on miR-34 family members showed that besides miR-34a-5p, its complementary strand miR-34a-3p was also upregulated in both, EVs and PBMCs after exposure to ionizing radiation, with fold changes similar to those of miR-34a-5p (Supplementary Fig. 3). Besides miR-34a, no other miR-34 family members were upregulated in tEVs (Supplementary Fig. 3 A), whereas miR-34a-3p and miR-34b-5p were significantly upregulated in sEVs (Supplementary Fig. 3 B). For sEVs miR-34c-5p and for lEVs all miR-34 family members except miR-34a-5p were below the detection level.

**Fig. 3:**
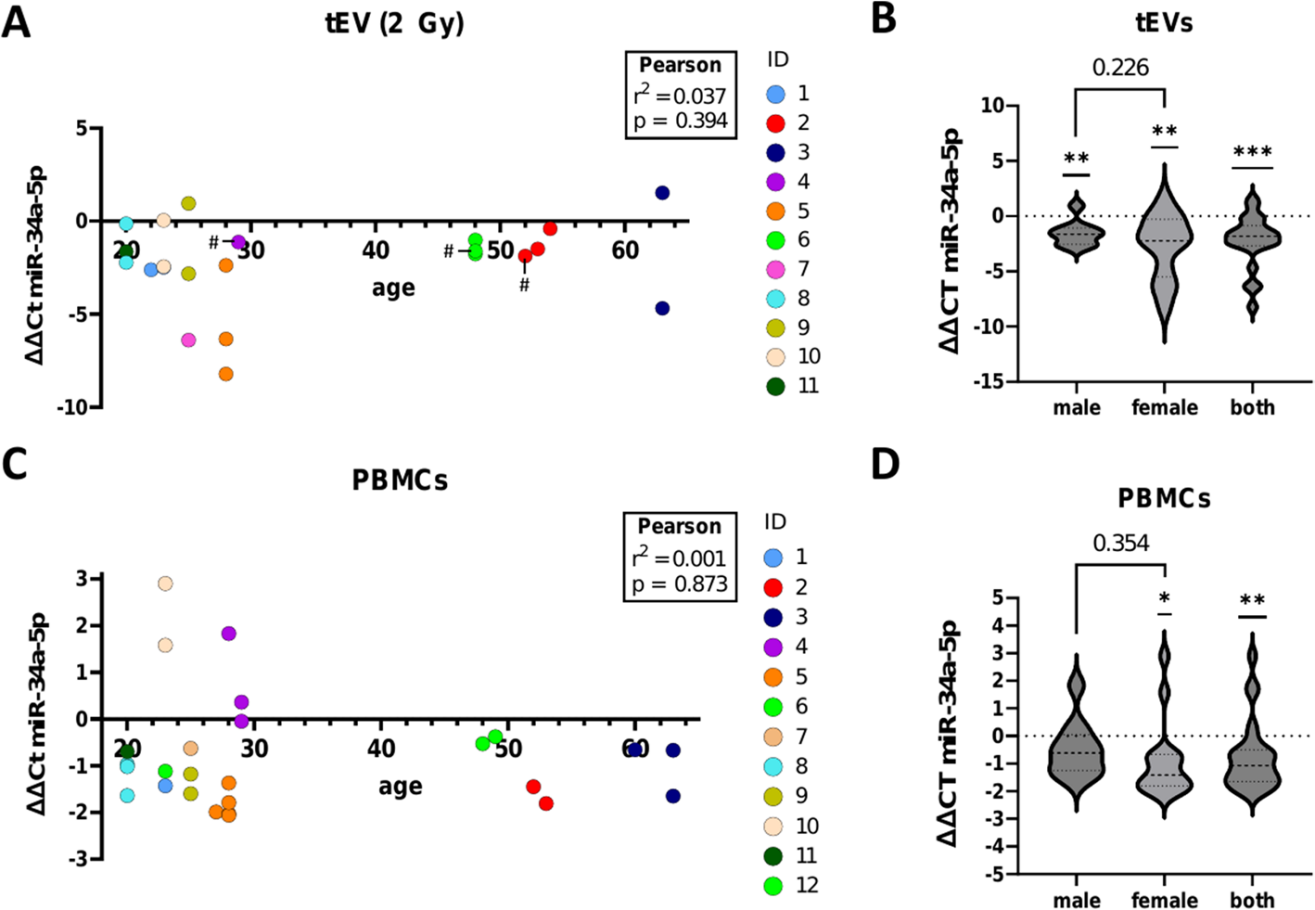
Individual miR-34a-5p radiation-induced regulation. ΔΔCt values of miR-34a-5p expression changes in tEVs isolated by PEG and UC (marked with #) (**A**) (**B**) or PBMCs (**C**) (**D**) after whole blood irradiation with 2 Gy. 22 tEV samples from 5 male (9 samples) and 6 female (13 samples) donors and 26 PBMC samples from 5 male (10 samples) and 7 female (16 samples) donors were investigated. Identically colored data points represent samples from the same donor taken at different time points. Data are shown as ΔΔCt values instead of fold-changes for better data visualization due to the high interindividual differences. For statistical analysis, Pearson correlation (age) or one-sample and student’s t-test (sex) have been performed

To investigate potential individual differences in radiation-induced upregulation of miR-34a-5p, we included additional donors and also examined intraindividual variations. The examination of miR-34a-5p levels in tEVs and their corresponding PBMCs across eleven donors (5 male and 6 female for tEVs, 5 male and 7 female for PBMCs, aged 20 to 63 years) showed interindividual variance (Fig. 3A, C). Furthermore, intraindividual variances were found in blood samples collected at distinct time points from identical donors. While no statistically significant differences were found for age or sex, an overall upregulation of miR-34a-5p was observed across all samples and donors following exposure to 2 Gy (Fig. 3B, D).

Together, these results showed that miR-34a-5p is significantly upregulated in a dose-dependent manner within total EVs (tEVs) in a broad range of donors and particularly in small EVs (sEVs) following ionizing radiation, highlighting its potential as a biomarker for radiation exposure, while other tested miRNAs showed minimal radiation-induced changes.

### Radiation-induced increase of EV release and miR-34a-5p levels are ATM-dependent

PBMCs were extracted from whole blood and exposed to 1, 2, and 4 Gy or sham-irradiation. After a period of 24–96 h, tEVs were isolated and analyzed by NTA to assess the amount and size of particles. Although cells release different types of vesicles, we focused on tEVs to gain a general overview of the effects of radiation on vesicle release.

**Fig. 4:**
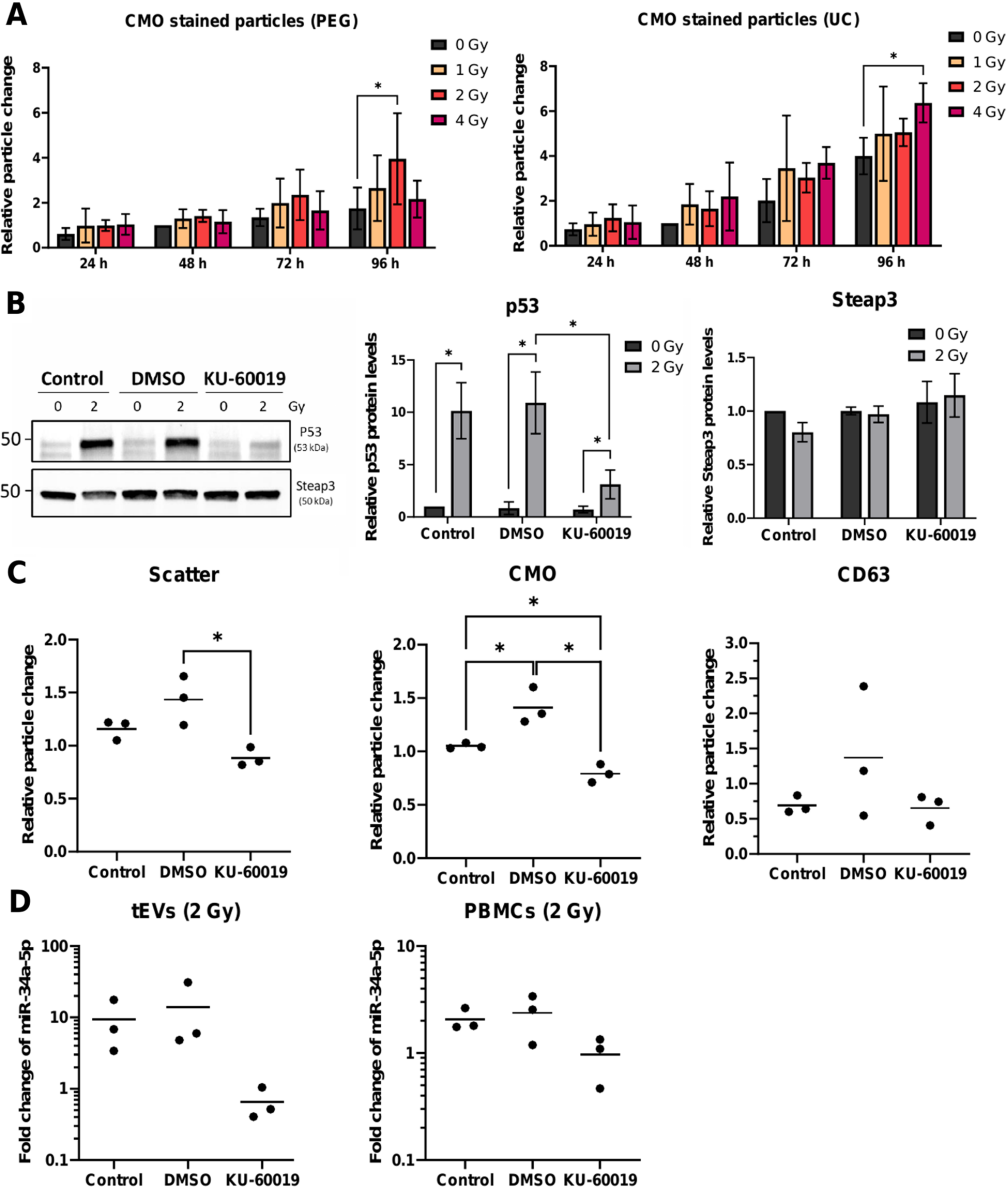
EV release from PBMCs after irradiation and the significance of ATM for the radiation-induced changes in EVs of PBMCs. **A** Relative particle change of tEV from PBMCs isolated by PEG precipitation or UC 24 – 96 h after irradiation. The concentration of particles in the tEV isolates was determined after staining with the cell membrane stain CellMask Orange (CMO) and normalized to particle concentrations of sham-irradiated samples at 48 h as an intermediate time point. **B** Western Blot of PBMCs 24 h after 2 Gy irradiation in the presence of 3 µM of the ATM-Inhibitor KU-60019 or 0.01% DMSO (solvent control). Relative protein levels of p53 and Steap3 normalized to total protein levels determined with the stain-free method are shown. **C** Relative particle change and **D** miR-34a-5p expression in tEVs derived from PBMCs or cellular miR-34a-5p levels 96 h after irradiation in the presence of the ATM-Inhibitor KU-60019. Mean values ± SD or single data points of three biological replicates are shown. For statistical analysis, a two-way ANOVA and multiple comparisons to 0 Gy were performed using Dunnett’s multiple comparison test or one-sample t-tests and multiple unpaired t-tests with Benjamini–Hochberg correction

Regardless of the isolation method, a time-dependent increase in the number of EVs was observed (Fig. 4 A, Supplementary Fig. 4 A), demonstrating a continuous release of vesicles from PBMCs over time (Supplementary Fig. 4 B). The measurements also indicated a trend towards an elevated release of tEVs following irradiation. However, this increase was only significant after 96 h. Considering the decrease in viable cells with increasing dose and time after irradiation, the increase in EV release in response to radiation becomes even more apparent when the particle changes are normalized to the surviving cells (Supplementary Fig. 4 C, D). Regarding the effect of ionizing radiation on EV size, no significant changes were observed (Supplementary Table 3).

To investigate whether the radiation-induced increase in vesicle release and miR-34a-5p upregulation is dependent on ATM, ATM activity was blocked using the highly specific small molecule inhibitor KU-60019 [26]. The inhibitory effect of KU-60019 on ATM activation was verified by the suppressed induction of the downstream target p53 in response to irradiation (Fig. 4B). However, the expression of the p53 downstream protein Six-Transmembrane Epithelial Antigen of Prostate 3 (Steap3), which has been shown in other studies to be associated with increased vesicle release after irradiation [27], remained unchanged 24 h (Fig. 4B) and 96 h (Supplementary Fig. 5 A) after irradiation.

NTA analysis of EVs revealed that while the release of tEVs in the DMSO control group slightly increased in all samples following irradiation, the release did not increase or was even reduced in the presence of the ATM inhibitor (Fig. 4C). In contrast, ATM inhibition did not affect the EV size (Supplementary Fig. 5 B). Additionally, ATM inhibition prevented the increase of miR-34a-5p in the tEVs and PBMCs after radiation exposure (Fig. 4D).

### Keratinocytes and fibroblasts strongly interact with EVs of PBMCs

To explore the biological functions of PBMC-derived EVs, the interaction/uptake of fluorescence-labeled tEVs with different potential recipient cell types was investigated after co-culture for 24 h. Since biological effects were described for various EVs encompassing a wide size range including small and large vesicles (e.g. exosomes, microvesicles, and apoptotic bodies) [28], total EVs rather than specific subpopulations were investigated.

**Fig. 5:**
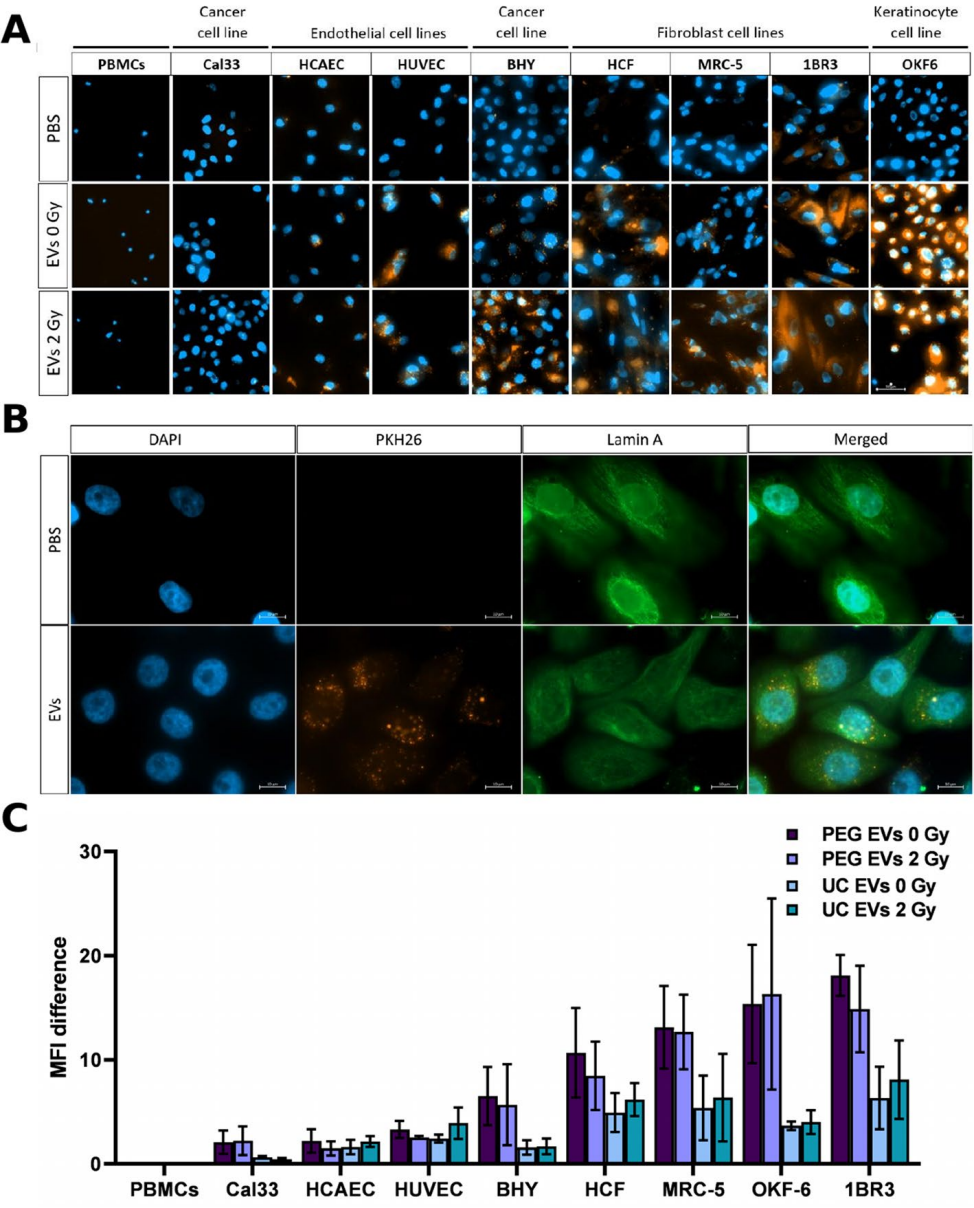
Interaction of PBMC-derived EVs with different cell lines. **A** Representative images of different recipient cell lines (200 × magnification) and **B**) high-resolution images of OKF6 (1000 × magnification) 24 h after co-cultivation with PKH26-labeled tEVs of PBMCs isolated by PEG precipitation or PBS. The nuclei of the cells are marked in blue (DAPI). EVs appear in orange (PKH26) and the cytoskeleton is stained in green (Lamin A). Scale bars represent A) 50 µm and B) 10 µm. **C** Mean fluorescence intensity (MFI) of the different cell types was measured using FACS analysis 24 h after co-cultivation with PKH26-labeled tEVs of 2 Gy or sham-irradiated PBMCs, or PBS control. Mean values ± SD of at least three biological replicates are shown

Both EV isolation methods revealed consistent cell type-dependent patterns in EV-cell interactions (Fig. 5A, C). The highest interactions were observed with keratinocytes (OKF6) and fibroblasts (1BR3). Endothelial cells and tumor cell lines showed moderate levels of fluorescence, whereas PBMCs displayed very low levels of fluorescence. High-resolution z-stack images of OKF6 revealed that the EV signal was not only distributed on the cell surface but throughout the entire cell (Fig. 5B). Irradiation of the donor PBMCs did not alter the EV-cell interaction. The EV isolation method, on the other hand, had a strong effect on EV-cell interactions. EVs isolated by UC yielded about half of the signal intensity compared to EVs isolated by PEG precipitation (Fig. 5C).

### miR-34a-5p decreases viability and induces senescence

Since miR-34a-5p levels were found to increase in EVs from irradiated PBMCs, the functions of miR-34a-5p were tested on the recipient cells OKF6, 1BR3, and HCAECs by miR-34a-5p mimic transfection. OKF6 and 1BR3 were chosen as they showed high interactions with PBMC-derived EVs, whereas HCAECs represent endothelial cells that are in direct contact with PBMC EVs in vivo.

So far, over 700 experimentally validated mRNA targets and over 2700 pathways have been reported for miR-34a-5p in the miRTarBase [29] and miRPathDB v2.0 [30]. Amongst the most significant pathways are apoptosis and cellular senescence. Especially the miR-34a targets B-cell lymphoma 2 (BCL-2) and Sirtuin-1 (SIRT1) have been shown to be important mediators of these pathways in response to ionizing radiation and their downregulation has been shown to enhance radiosensitivity [31–33].

Overexpression of miR-34a-5p in the transfected cell lines was confirmed by qPCR in all three cell lines (Supplementary Fig. 6 A). However, expression analysis of miR-34a-5p targets by qPCR revealed cell type-specific differences. While *SIRT1* and *BCL-2* are successfully reduced by miR-34a-5p overexpression in OKF6, only *BCL-2* is downregulated in HCAEC, whereas *SIRT1* is reduced in 1BR3 (Supplementary Fig. 6 B-D).

**Fig. 6:**
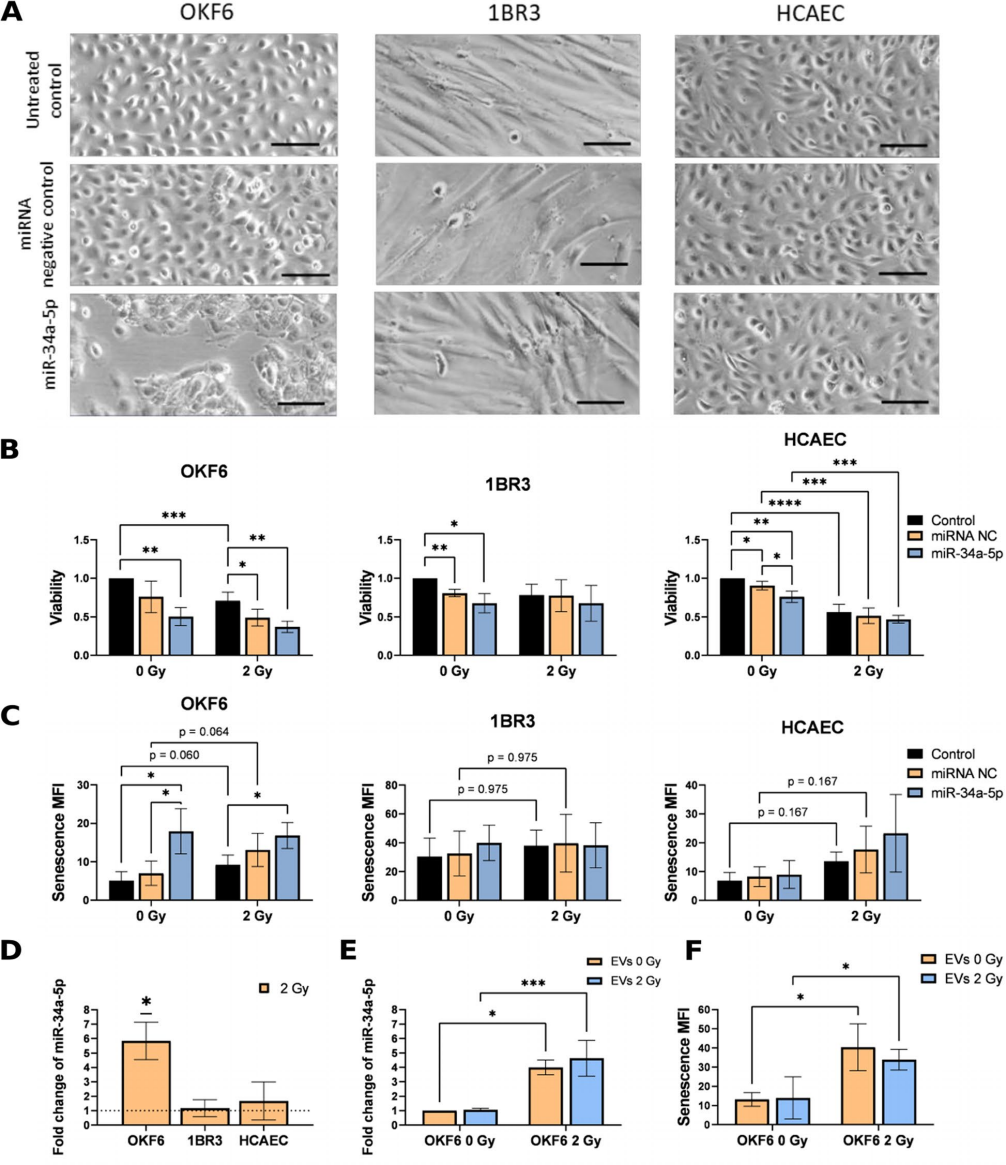
Effect of miR-34a-5p on recipient cells. **A** Microscopic images, **B** Viability, and **C** Senescence of OKF6, 1BR3, and HCAECs 72 h after transfection with 10 nm of a miRNA negative control or 10 nm miR-34a-5p. After 1 h incubation, cells were irradiated with 0 or 2 Gy. The scale bar represents 100 µm. Mean values ± SD of five biological replicates are shown. **D** Mir-34a-5p upregulation in OKF6, 1BR3, and HCAECs 72 h after 2 Gy irradiation. **E** Mir-34a-5p expression and **F** senescence in OKF6 72 h after adding PEG isolated EVs of 0 and 2 Gy irradiated PBMCs. For statistical analysis, 2-way ANOVA and multiple t-tests with Benjamini–Hochberg correction (B, C, E, F) or one-sample t-tests (D) were performed

To investigate the biological consequences of increased miR-34a-5p levels, the three cell lines were assessed for morphology, viability, senescence, and apoptosis 72 h after miR-34a-5p mimic transfection (Fig. 6, Supplementary Fig. 7).

Microscopic images of miR-34a-5p transfected OKF6 cells revealed notable changes in cell shape and size. MiR-34a-5p transfection led to an increase in cell size and a flattened cell morphology compared to cells transfected with an unspecific control miRNA (Fig. 6 A), indicating a phenotype typical of senescence [34]. In contrast, the fibroblast cell line 1BR3 and the endothelial cell line HCAECs did not show any morphological changes.

Cell viability was measured by metabolic activity using a WST assay. Figure 6B demonstrates that miR-34a-5p transfection led to a cell type-specific decrease in viability in sham-irradiated cells compared to the transfection with a control miRNA (significant in HCAECs). In irradiated cells, the contribution of miR-34a-5p to the decrease in viability compared to the transfection of NC miRNA was less pronounced.

Senescence was analyzed 72 h after irradiation using flow cytometry for beta-galactosidase (SA-β-gal) activity. In accordance with the morphological changes, the most pronounced effects were observed in the OKF6 cell line (Fig. 6C). A statistically significant increase in the mean fluorescence intensity (MFI) of SA-β-gal-positive cells in miR-34a-5p-transfected, unirradiated OKF6 cells in comparison to cells transfected with NC miRNA or untreated cells, demonstrated the induction of senescence by miR-34a-5p. Additional irradiation did not further increase senescence in miR-34a-5p transfected OKF6 cells.

However, irradiation increased senescence in non-tansfected and control miRNA-transfected OKF6 (Fig. 6C and F). Furthermore, irradiation of the OKF6 cells significantly increased cellular miR-34a-5p levels (Fig. 6D). In contrast, miR-34a-5p transfection did not induce senescence in fibroblasts and endothelial cells. Also, irradiation did not affect miR-34a-5p expression and senescence induction in these cells (Fig. 6C and D).

The impact of miR-34a-5p overexpression on apoptosis was also tested by quantifying phosphatidylserine on the membrane surface using Annexin V staining (Supplementary Fig. 7). Transfection itself led to an increase in apoptotic OKF6 cells, however, miR-34a-5p did not further increase apoptosis. In 1BR3 and HCAEC cells, a small increase in apoptotic cells was observed.

To assess, whether EVs from irradiated PBMCs could induce miR-34a-5p in recipient cells and lead to functional changes in senescence, the keratinocytes OKF6 were co-cultivated for 72 h with tEVs from 2 Gy or sham irradiated PBMCs isolated by PEG precipitation. In this setting, miR-34a-5p levels (Fig. 6E) and the MFI of senescent cells (Fig. 6F) remained unchanged in recipient cells, receiving EVs from 2 Gy or sham-irradiated PBMC. But again a significant upregulation of miR-34a-5p and induction of senescence could be observed after irradiation.

## Discussion

EVs play a crucial role in facilitating cell–cell communication processes responsible for systemic radiation effects. This study provides valuable insights into radiation-induced miRNA changes in EVs from blood cells and has identified keratinocytes and fibroblasts to highly interact with EVs. It was shown that miR-34a-5p, which is highly increased in EVs after irradiation, led to a cell type-dependent decrease in viability and induction in senescence.

### Radiation increased the miR-34a-5p content in released EVs

To identify radiation effects on EVs secreted from PBMCs, ex vivo-irradiated blood was used as an exposure model, which was demonstrated to accurately reflect the in vivo peripheral blood radiation response in humans [35]. To account for different types of EVs and to identify potential differences resulting from EV isolation methods, the findings were validated in EV subfractions (total EVs, large EVs, and small EVs) by applying two different isolation methods.

For both, tEVs and sEVs higher yields were observed after PEG precipitation compared to UC. This was equally shown by NTA using light scatter, membrane-staining, and detection of CD63. However, both methods resulted in the isolation of vesicles with similar diameters.

Time course analysis showed a constant release of EVs by PBMCs over time and tEV release additionally increased with the irradiation dose. Recent publications using lung cancer cell lines or mouse models suggest that stress-induced increases in EV release are mediated by increased *STEAP3* transcription, which in turn was induced by p53 [27, 36, 37]. However, our data did not confirm this model for tEVs in PBMCs, because the protein levels of STEAP3 remained unchanged at 24 h and 96 h after radiation although p53 was induced. Also, inhibition of p53 accumulation by a small molecule ATM inhibitor does not affect tEV release in response to radiation. Therefore, alternative mechanisms may account for radiation-increased EV release in PBMCs. A missing connection between p53 and STEAP3 expression was also found in cancer-associated fibroblasts and colon cancer cells [38, 39], and alternative pathways involving proteins like ARF6, RAB27a, and RAB27b may contribute to the regulation of EV secretion [40, 41].

Among all EV cargo components, miRNAs are particularly interesting candidates, as they can regulate gene expression in recipient cells [42]. Several miRNAs were previously found to be radiation-responsive in sEVs from PBMCs in a pooled analysis of four female donors [10]. In a more holistic approach, this study included total, small, and large EV fractions in individual male and female donors within the wide age group of 20 to 63 years.

An upregulation of miR-34a-5p in 19 out of 22 tEV preparations from 11 individual donors was confirmed, despite interindividual differences and differences between samples obtained from the same donor in a period of 2 years. This finding further emphasized increased vesicle-mediated release of miR-34a-5p as a general and reproducible radiation response mechanism. Although sex-specific differences were reported for vesicle loading [43], neither age- nor sex-specific differences could be found in this study. Even though a dose-dependent increase of miR-34a-5p in both EVs and PBMCs could be observed, expression levels of miR-34a-5p were not correlated in vesicles and EV donor cells. Several studies made similar observations, showing that cellular miRNA profiles differ significantly from those in EVs [44]. Furthermore, similar miRNA profiles in EVs of different cell types indicate that specific sorting mechanisms for miRNA loading into EVs may be responsible for these differences. While increased cellular miR-34a-5p expression following irradiation has been found before in other cell systems including lymphocytes after whole blood irradiation [45–48], our results showed that this upregulation persisted up to 96 h after irradiation. In parallel to miR-34a-5p, Salzman et al. [49] reported the radiation-induced upregulation of all miR-34 family members in lung cancer cells in an ATM-dependent manner. Accordingly, our results showed abolished miR-34a-5p upregulation in PBMCs and tEVs when p53 stabilization is blocked by an ATM inhibitor. However, the other miR-family members miR-34b/c were not significantly upregulated after radiation exposure, suggesting additional regulatory mechanisms for miR-34 family members.

Comparisons of sEVs and lEVs revealed differences in terms of particle size and composition. On the one hand, the differences in particle sizes indicate different proportions of small and large vesicles such as exosomes and microvesicles in the EV fractions. On the other hand, CD63-positive particles were enriched in sEV samples and significantly smaller than unstained or membrane-stained particles. Previous publications suggest that CD63 is abundant in endosomes and therefore is often considered as an exosome marker, whereas it is present in microvesicles to a lesser extent [50]. Additionally, the miRNA composition differed between sEVs and lEVs, as changes in miR-106a-5p, miR-155-5p, and miR-451a-5p expression levels could exclusively be detected in small EVs, whereas only miR-34a-5p was also increased in large EVs. Additionally, miR-34a-5p upregulation was more pronounced in sEVs than in tEVs or lEVs, suggesting that sEVs are highly radiation-controlled vehicles for the release of miRNAs.

The identification of additional radiation-responsive miRNAs enhances the potential of sEVs as versatile biomarkers for radiation exposure. As a further advantage, this study revealed a sustained increase of EV-derived miRNAs persisting for a minimum of 4 days. This is in contrast to several currently suggested biomarkers that hinge on gene or protein expression, such as gamma-H2AX foci or FDXR, which quickly return to baseline levels [47, 48].

### EVs from PBMCs showed pronounced uptake by keratinocytes and fibroblasts

Potential cell type specificity in EV uptake is a critical issue in understanding their biological functions. Previous studies have reported that EVs derived from different cell types preferentially bind to specific recipient cells [51, 52]. Our results showed significant differences in the uptake efficiency of identical EV preparations in different cell types. Keratinocytes and fibroblasts were identified as preferred recipients while PBMCs take up only minor amounts regardless of a previous irradiation of the donor cells. The underlying mechanism for these differences is not clear yet. Different uptake mechanisms, the cellular environment, and specific surface properties of EVs and/or recipient cells, such as surface proteins and lipids may account for this observation [51, 53]. Low uptake of PBMC-derived EVs in lymphocytes was also found in previous studies [10, 54]. Together, these results suggest a targeted communication of PBMC-derived EVs with specific recipient cells rather than random interactions.

Interestingly, co-cultivation with equal concentrations of EVs from PEG precipitation results in a more pronounced uptake than for EVs from UC isolation. One possible explanation is that PEG may impact EV binding capacity by preventing EV aggregation [55]. Alternatively, the centrifugal force used in UC may compromise the integrity of the EV membrane. In this regard, several studies have shown that UC and size exclusion chromatography can result in a loss of corona proteins around EVs [56], which can lead to functional impairments [57].

### miR-34a-5p affects viability and senescence in recipient cells

MiR-34a-5p functions were analyzed in fibroblasts, keratinocytes, and endothelial cells. The documented functions of miR-34a-5p were predominantly related to its role as a tumor suppressor. Notable effects include decreased proliferation, survival, and migration, along with an observed increase in apoptosis [58–60].

Additionally, the involvement of miR-34a-5p in senescence has been described in cancer and non-cancer cells. For example, miR-34a-5p functions have been reported in age-related vascular and neurodegenerative diseases [61, 62]. In keratinocytes, elevated miR-34a-5p impaired wound healing and increased inflammatory response [63].

MiRNA mimic transfection resulted in reduced viability in endothelial cells. Additionally, an increase in senescence could be observed in OKF6 in response to miR-34a-5p mimic transfection, along with the downregulation of the anti-senescence protein SIRT1, supporting the potential senescence-promoting function of miR-34a-5p. Furthermore, ionizing radiation demonstrated elevated levels of miR-34a-5p as well as a trend towards increased numbers of senescent cells in control and NC RNA transfected OKF6 (p = 0,06 and 0,064). Induction of senescence by ionizing radiation was also observed in stem cell-derived keratinocytes [64] and epidermal skin keratinocytes [65]. Together, these findings imply that the induction of senescence in keratinocytes by ionizing radiation could potentially be associated with an increase in miR-34a-5p levels as suggested for lung cancer cells [66]. However, to definitively establish a direct causal relationship, further experiments are required. For example, blocking the radiation-induced increase in miR-34a-5p through antagomir transfection and assessing its impact on senescence levels could be used to confirm this interdependency. Notably, miR-34a-5p effects were less evident in fibroblasts and endothelial cells suggesting cell-type specific miR-34a-5p functions.

Several studies describe the regulatory effects of EV-derived miRNAs on recipient cells [42, 67, 68]. In parallel it was shown that EVs play an important role in radiation-induced cell communication [69]. Applying these findings to our results, biological effects of EVs through the transfer of miR-34a-5p to keratinocytes were expected (Supplementary Fig. 8). However, the initial experimental setup did not confirm an EV-induced effect on recipient cells. Even though transfection with miR-34a-5p resulted in a strong induction of senescence in keratinocytes, no differences in senescence in the recipient cells could be detected after co-cultivation with EVs from irradiated PBMCs with increased miR-34a-5p content. In future experiments, a systematic co-cultivation using various time points and EV concentrations may challenge this result.

This study provides valuable new insights into the radiation response of PBMCs. However, several limitations constrain the strength of these findings. A primary limitation is the relatively small sample size, which was restricted by the logistical challenges of isolating sufficient PBMCs from donors. Additionally, significant interindividual variability in donor responses to irradiation was observed, potentially contributing to large variances and, in some cases, preventing trends from reaching statistical significance. A larger sample size could help address these issues by reducing variability and generating more robust data. Future studies with larger cohorts could better account for individual differences in PBMC responses and minimize inconsistencies.

## Conclusion

Understanding the implications of EV-mediated intercellular communication in the context of radiation exposure holds promise for the development of strategies to mitigate the side effects of radiotherapy on healthy tissues. This study demonstrated the effects of ionizing radiation on extracellular vesicles from PBMCs in terms of uptake, cargo, and release. We were able to show that regardless of irradiation, PBMC-derived EVs preferentially interacted with fibroblasts and keratinocytes. The most notable radiation-induced miRNA cargo alteration was observed in sEVs for miR-34a-5p, highlighting their potential as biomarker candidates that remain detectable even at later stages following exposure to ionizing radiation. Functional analysis of this miRNA revealed a cell type-specific enhancement in senescence and a decrease in viability.

Nonetheless, the direct involvement of EVs from PBMCs in the biological processes of recipient cells needs to be further analyzed. Given the established role of miR-34a-5p in cardiovascular and neurodegenerative diseases, and considering that cardiovascular diseases and neurological impairments are recognized side effects of radiotherapy, it is advisable to prioritize investigations in these target tissues.

## Supplementary Information

Additional file 1: Supplementary Table 1. miRNA and mRNA primers used for qPCR analysis. Supplementary Table 2. Primary antibodies for western blot analysis. Supplementary Table 3. EV sizes after ionizing radiation. Supplementary Fig. 1. Size distribution of PBMC-derived sEVs and lEVs. Supplementary Fig. 2. Correlation of miR-34a-5p expression. Supplementary Fig. 3. miR-34 family member expression in response to ionizing radiation. Supplementary Fig. 4. EV release from PBMCs after irradiation. Supplementary Fig. 5. Significance of ATM for the radiation-induced changes in EVs of PBMCs. Supplementary Fig. 6. Expression analysis in miR-34a-5p transfected recipient cells. Supplementary Fig. 7. Apoptosis in miR 34a 5p transfected recipient cells. Supplementary Fig. 8. Suggested model for the EV-based communication between PBMCs and keratinocytes with focus on miR-34a-5p.

Additional file 2. Raw images of western blots.

## Data Availability

The datasets generated during and/or analyzed during the current study are available from the corresponding author upon reasonable request.

## Abbreviations

ATM: Ataxia Teleangiectasia Mutated
BSA: Bovine Serum Albumin
CMO: CellMask Orange
DMSO: Dimethyl sulfoxide
DMEM: Dulbecco´s Modified Eagle Medium
DPBS: Dulbecco´s Phosphate Buffered Saline
DTT: Dithiothreitol
EDTA: Ethylenediaminetetraacetic acid
EMEM: Eagle's minimum essential medium
EVs: Extracellular vesicles
ER: Endoplasmatic reticulum
FACS: Fluorescence Activated Cell Sorting
FBS: Fetal bovine serum
Gy: Gray
HCAEC: Human coronary artery endothelial cells
HCF: Human coronary fibroblasts
HUVEC: Human umbilical vein endothelial cells
lEVs: Large extracellular vesicles
MFI: Mean fluorescence intensity
NK cells: Natural killer cells
NTA: Nanoparticle tracking analysis
PBMCs: Peripheral blood mononuclear cells
PEG: Polyethylene glycol
PFA: Paraformaldehyde
PVDF: Polyvinylidenfluorid
qPCR: Quantitative real-time polymerase chain reaction
RIBE: Radiation-induced bystander effect
SDS: Sodium dodecyl sulfate
sEVs: Small extracellular vesicles
TBS-T: Tris-buffered saline with Tween20
tEVs: Total extracellular vesicles
UC: Ultracentrifugation
WST: Water-soluble tetrazolium

## Data availability

All data supporting the findings of this study are available within the paper and its Supplementary Information.
