# Supplementary material for: Ionizing radiation effects on blood-derived extracellular vesicles: insights into miR-34a-5p-mediated cellular responses and biomarker potential
Source: Cell Commun Signal. 2024 Oct 2;22:471. doi: 10.1186/s12964-024-01845-x (PMC11446100; doi:10.1186/s12964-024-01845-x)
Supplement: Supplementary file 1 — Additional file 1: Supplementary Table 1. miRNA and mRNA primers used for qPCR analysis. Supplementary Table 2. Primary antibodies for western blot analysis. Supplementary Table 3. EV sizes after ionizing radiation. Supplementary Fig. 1. Size distribution of PBMC-derived sEVs and lEVs. Supplementary Fig. 2. Correlation of miR-34a-5p expression. Supplementary Fig. 3. miR-34 family member expression in response to ionizing radiation. Supplementary Fig. 4. EV release from PBMCs after irradiation. Supplementary Fig. 5. Significance of ATM for the radiation-induced changes in EVs of PBMCs. Supplementary Fig. 6. Expression analysis in miR-34a-5p transfected recipient cells. Supplementary Fig. 7. Apoptosis in miR 34a 5p transfected recipient cells. Supplementary Fig. 8. Suggested model for the EV-based communication between PBMCs and keratinocytes with focus on miR-34a-5p. [file 12964_2024_1845_MOESM1_ESM.docx]

1. Supplementary material

**Supplementary table 1: miRNA and mRNA primers used for qPCR analysis**

| **Target** | **Accession Number/Oligonucleotide sequence** | **Supplier** |
| --- | --- | --- |
| **miRNA** | | |
| hsa‑miR‑16‑5p | YP00205702 | Qiagen miRCURY LNA miRNA PCR Assays |
| hsa‑miR‑34a‑5p | YP00204486 |  |
| hsa‑miR‑34a‑3p | YP00206061 |  |
| hsa‑miR‑34b‑5p | YP00204424 |  |
| hsa‑miR‑34b‑3p | YP00204005 |  |
| hsa‑miR‑34c‑5p | YP00205659 |  |
| hsa‑miR‑34c‑3p | YP00204373 |  |
| hsa‑miR‑106a‑5p | YP00204563 |  |
| hsa‑miR‑155‑5p | YP00204308 |  |
| hsa‑miR‑451a | YP02119305 |  |
| mRNA | | |
| BCL‑2 | Fwd: 5’ GATGTGATGCCTCTGCGAAG 3’ | Eurofins Genomics |
|  | Rv: 5’ CATGCTGATGTCTCTGGAATCT 3’ |  |
| SIRT1 | Fwd: 5’ TGCTGGCCTAATAGAGTGGCA 3’ |  |
|  | Rv: 5’ CTCAGCGCCATGGAAAATGT 3’ |  |
| Actin‑β | Hs_ACTB_1_SG, #QT00095431 | Qiagen QuantiTect Primer Assays |
| TBP1 | Hs_TBP_1_SG, #QT00000721 |  |
| GAPDH | Hs_GAPDH_1_SG, #QT00079247 |  |

**Supplementary table 2: Primary antibodies for western blot analysis**

| **Target protein** | **Dilution** | **Product number** | **Supplier** |
| --- | --- | --- | --- |
| Calnexin | 1:1000 | 2433S | Cell Signaling |
| TSG101 |  | GTX70255 | GeneTex, USA |
| Steap3 |  | ab151566 | Abcam, UK |
| p53 |  | ab1101 |  |
| CD81 | 1:200 | sc‑166029 | Santa Cruz, USA |
| ALIX | 1:1000 | VPA00765 | Bio‑Rad Laboratories, USA |
| Flotillin‑1 |  | VPA00126 |  |
| CD9 |  | MCA469GA |  |
| α‑Tubulin | 1:10000 | hFAB Rhodamine |  |

**Supplementary Table 3: Size of tEV released by PBMCs**. Median sizes of tEV from PBMCs isolated by PEG precipitation or UC 24 – 96 h after irradiation. The size of particles in the EV isolates were determined before (Scatter) and after staining with the cell membrane stain CellMask Orange (CMO). Mean values ± SD of three biological replicates are displayed.

| **Time point** | **Gy** | **Median size of particles ± SD [nm]** | | | | | | | | | | | | |
| --- | --- | --- | --- | --- | --- | --- | --- | --- | --- | --- | --- | --- | --- | --- |
|  |  | **Scatter** | | | | | | | **CMO** | | | | | |
|  |  | PEG | | | UC | | | | PEG | | | UC | | |
| **24 h** | **0** | 155 | ± | 27.0 | | 177 | ± | 8.0 | 183 | ± | 41.7 | 184 | ± | 19.3 |
|  | **1** | 152 | ± | 20.9 | | 180 | ± | 11.4 | 174 | ± | 32.5 | 215 | ± | 39.7 |
|  | **2** | 174 | ± | 6.5 | | 183 | ± | 4.8 | 199 | ± | 6.8 | 211 | ± | 28.9 |
|  | **4** | 170 | ± | 33.5 | | 183 | ± | 7.6 | 196 | ± | 46.1 | 221 | ± | 41.5 |
| **48 h** | **0** | 153 | ± | 19.2 | | 179 | ± | 6.8 | 178 | ± | 30.6 | 186 | ± | 7.4 |
|  | **1** | 149 | ± | 23.9 | | 175 | ± | 7.2 | 175 | ± | 44.3 | 206 | ± | 6.9 |
|  | **2** | 160 | ± | 19.6 | | 175 | ± | 5.1 | 201 | ± | 54.6 | 201 | ± | 6.5 |
|  | **4** | 157 | ± | 21.5 | | 183 | ± | 8.2 | 199 | ± | 58.6 | 212 | ± | 11.3 |
| **72 h** | **0** | 157 | ± | 12.4 | | 178 | ± | 16.4 | 191 | ± | 43.7 | 200 | ± | 24.8 |
|  | **1** | 158 | ± | 25.5 | | 182 | ± | 11.1 | 190 | ± | 46.2 | 219 | ± | 18.1 |
|  | **2** | 167 | ± | 20.2 | | 174 | ± | 5.7 | 195 | ± | 50.9 | 206 | ± | 21.8 |
|  | **4** | 161 | ± | 20.0 | | 193 | ± | 8.7 | 206 | ± | 43.6 | 215 | ± | 11.6 |
| **96 h** | **0** | 163 | ± | 12.7 | | 181 | ± | 17.7 | 196 | ± | 38.5 | 202 | ± | 22.0 |
|  | **1** | 162 | ± | 17.6 | | 187 | ± | 6.2 | 204 | ± | 30.3 | 216 | ± | 16.9 |
|  | **2** | 170 | ± | 6.3 | | 198 | ± | 6.9 | 213 | ± | 20.8 | 215 | ± | 18.1 |
|  | **4** | 163 | ± | 17.8 | | 188 | ± | 17.1 | 204 | ± | 42.9 | 210 | ± | 19.0 |


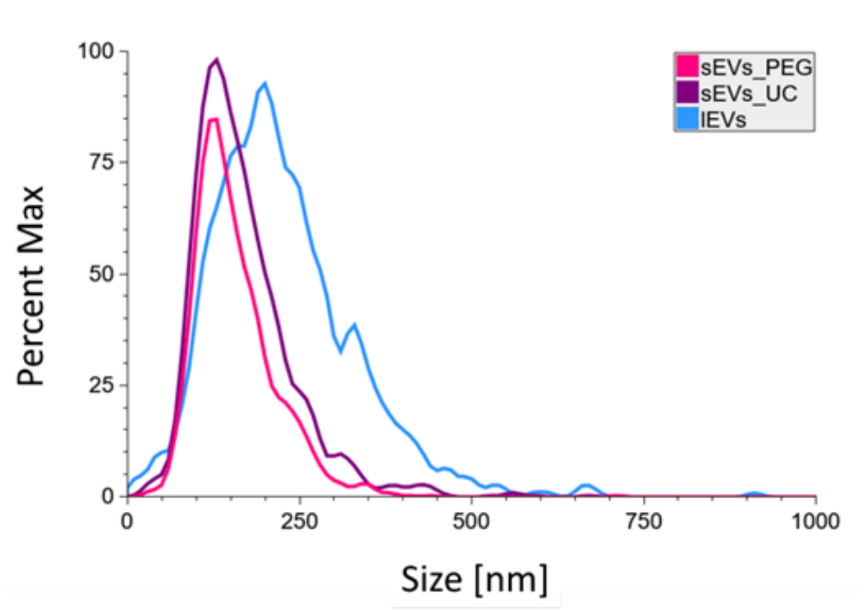


**Supplementary Fig. 1: Size distribution of PBMC‑derived sEVs and lEVs.** EVs were obtained 96 h after isolation of PBMCs. sEVs were isolated by PEG‑precipitation and UC. The size distribution of sEVs and lEVs was determined using NTA. A normalized histogram of a representative biological replicate was generated using the free web-based application floreada.io.


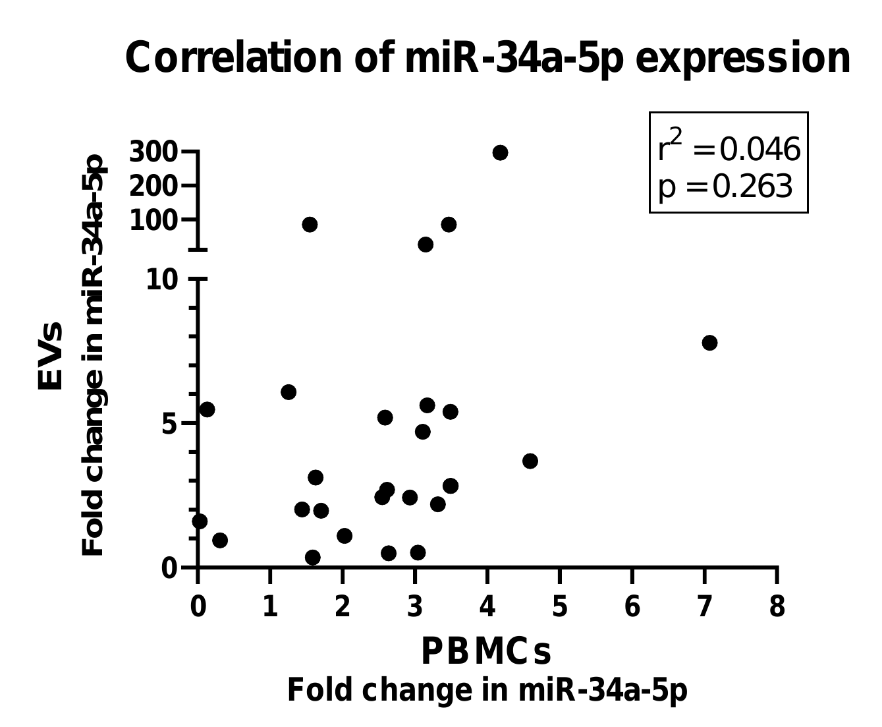


***Supplementary Fig. 2:*** *Correlation analysis of miR 34a 5p fold changes in tEVs (x axis) and corresponding PBMCs (y axis) after irradiation of whole blood. For correlation analysis, the Pearson correlation coefficient was calculated.*


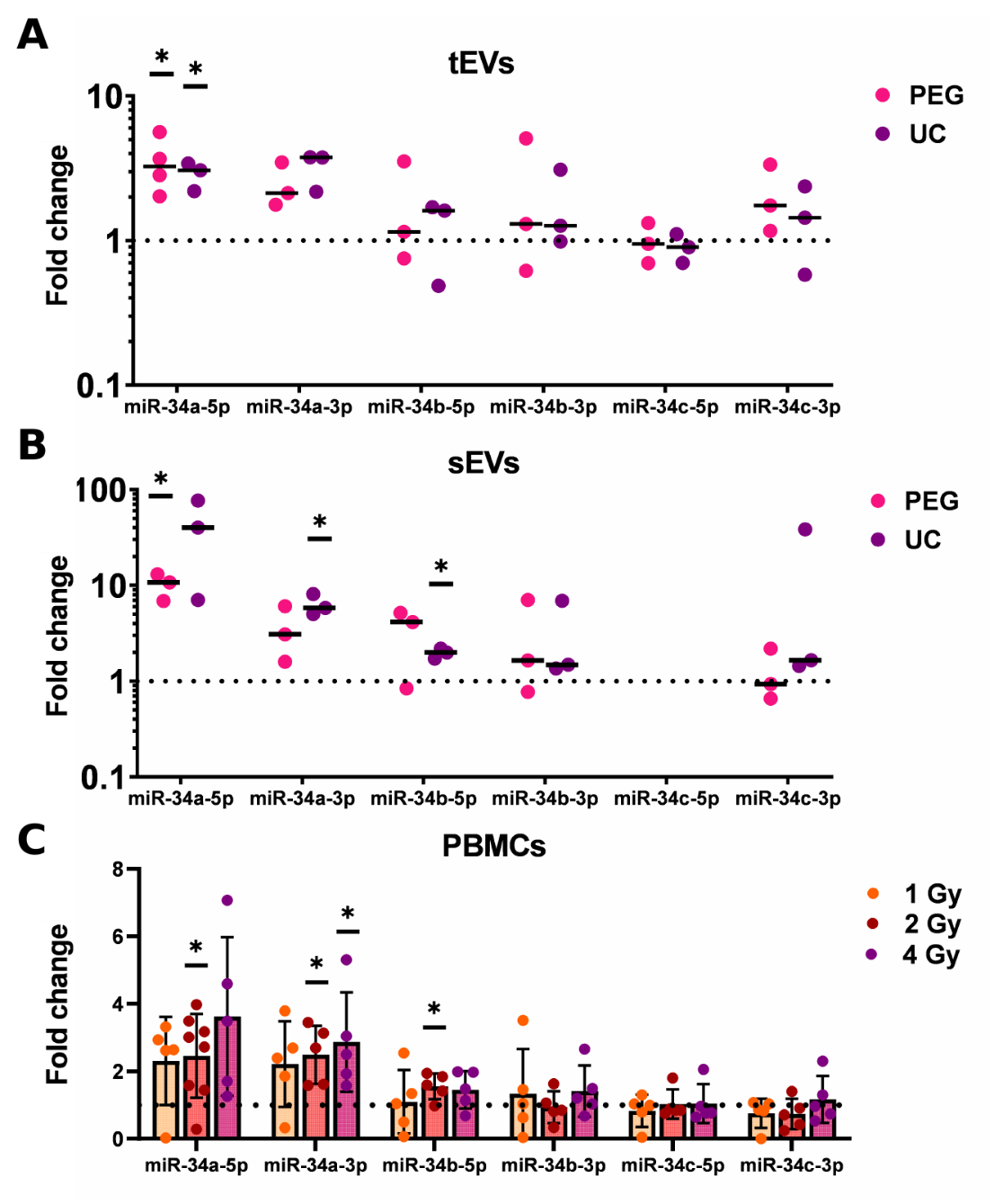


**Supplementary Fig. 3:** **miR‑34 family member expression in response to ionizing radiation**. Expression of miR‑34a/b/c in tEVs (A) and sEVs (B) of 2 Gy irradiated PBMCs isolated by PEG precipitation and corresponding cells (C) 96 h after irradiation of whole blood. Mean values ± SD of at least three (EVs) or five (PBMCs) biological replicates are shown. Statistical analysis was performed using one‑sample t‑tests: *p ≤ 0.05


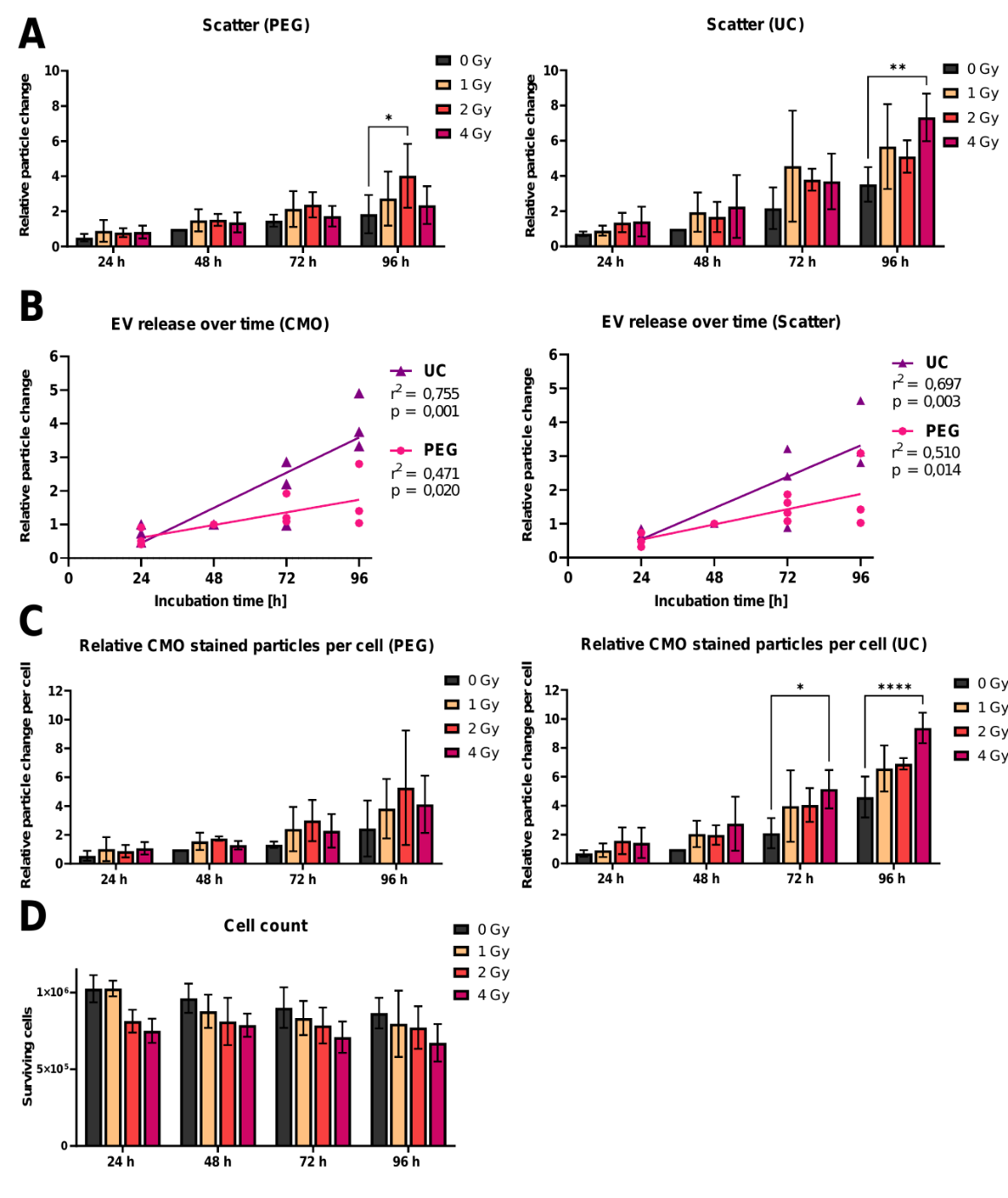


**Supplementary Fig. 4: EV release from PBMCs after irradiation.** A) Relative particle changes of tEV from PBMCs isolated by PEG precipitation or UC 24 – 96 h after irradiation. B) Linear regression of time dependent relative particle changes in tEVs of sham irradiated PBMCs. C) Relative particle changes of tEV from PBMCs isolated by PEG precipitation and UC 24 – 96 h after irradiation. C) Particle concentrations were normalized to the surviving cell counts, and the n-fold change was calculated. The concentration of particles in the tEV isolates was determined after staining with the cell membrane stain CellMask Orange (CMO) and normalized to particle concentrations of sham irradiated samples at 48 h as an intermediate time point. D) Surviving PBMCs were determined by trypan blue staining. Mean values ± SD of three biological replicates are shown. For statistical analysis, a two‑way ANOVA and multiple comparisons to 0 Gy were performed using Dunnett’s multiple comparison test.


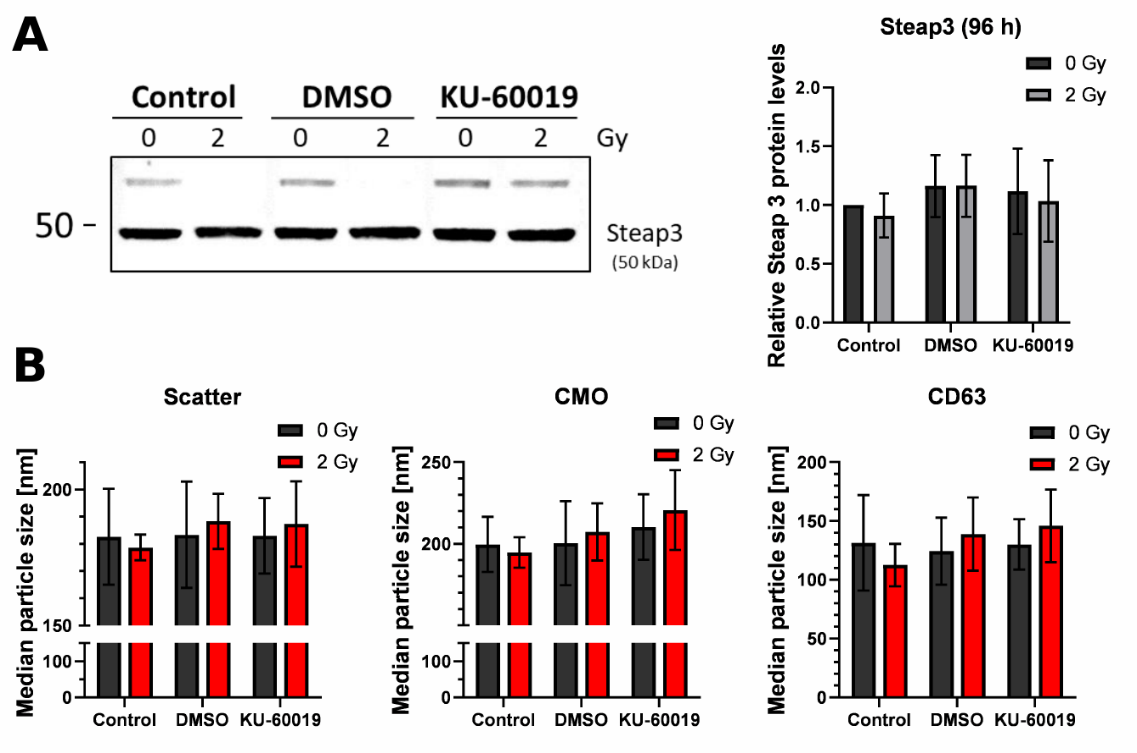


**Supplementary Fig. 5:** **Significance of ATM for the radiation‑induced changes in EVs of PBMCs** A) Western Blot of PBMCs and B) median particle size of tEVs derived from PBMCs 96 h after 2 Gy irradiation in the presence of 3 µM of the ATM‑Inhibitor KU‑60019 or 0.01% DMSO (solvent control). Relative protein levels of Steap3 normalized to total protein levels determined with the stain‑free method are shown. For NTA analysis, tEVs were isolated by UC after 96 h. Mean values ± SD of three biological replicates are shown. Statistical analysis was performed using and multiple t-tests with Benjamini-Hochberg correction.


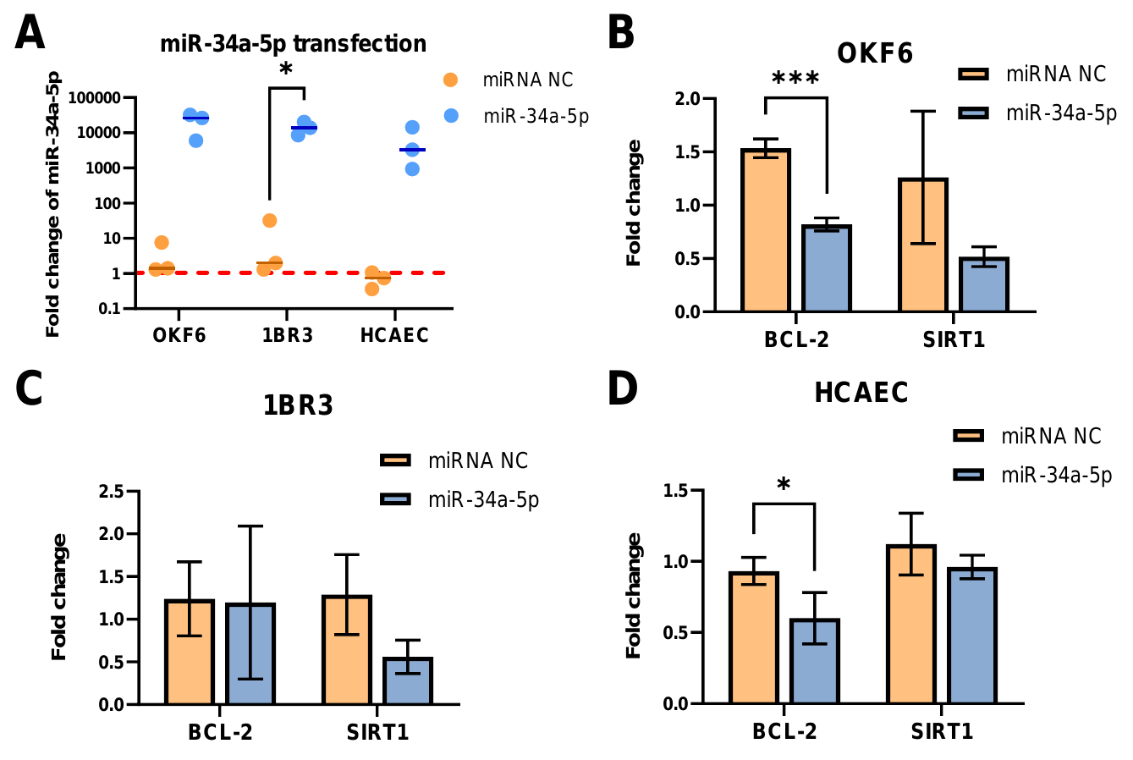


**Supplementary Fig. 6: Expression analysis in miR‑34a‑5p transfected recipient cells.** Expression of miR‑34a‑5p (A) and its direct targets BCL‑2 and SIRT1 48 h after transfection of B) OKF6 (n = 3), C) 1BR3 (n = 3) and D) HCAECs (n = 4) with 10 nm miR‑34a‑5p or a negative miRNA control. Expression levels were normalized to an untransfected control. For statistical analysis, multiple t-tests with Benjamini-Hochberg correction have been performed. *p ≤ 0.05 ***p ≤ 0.001


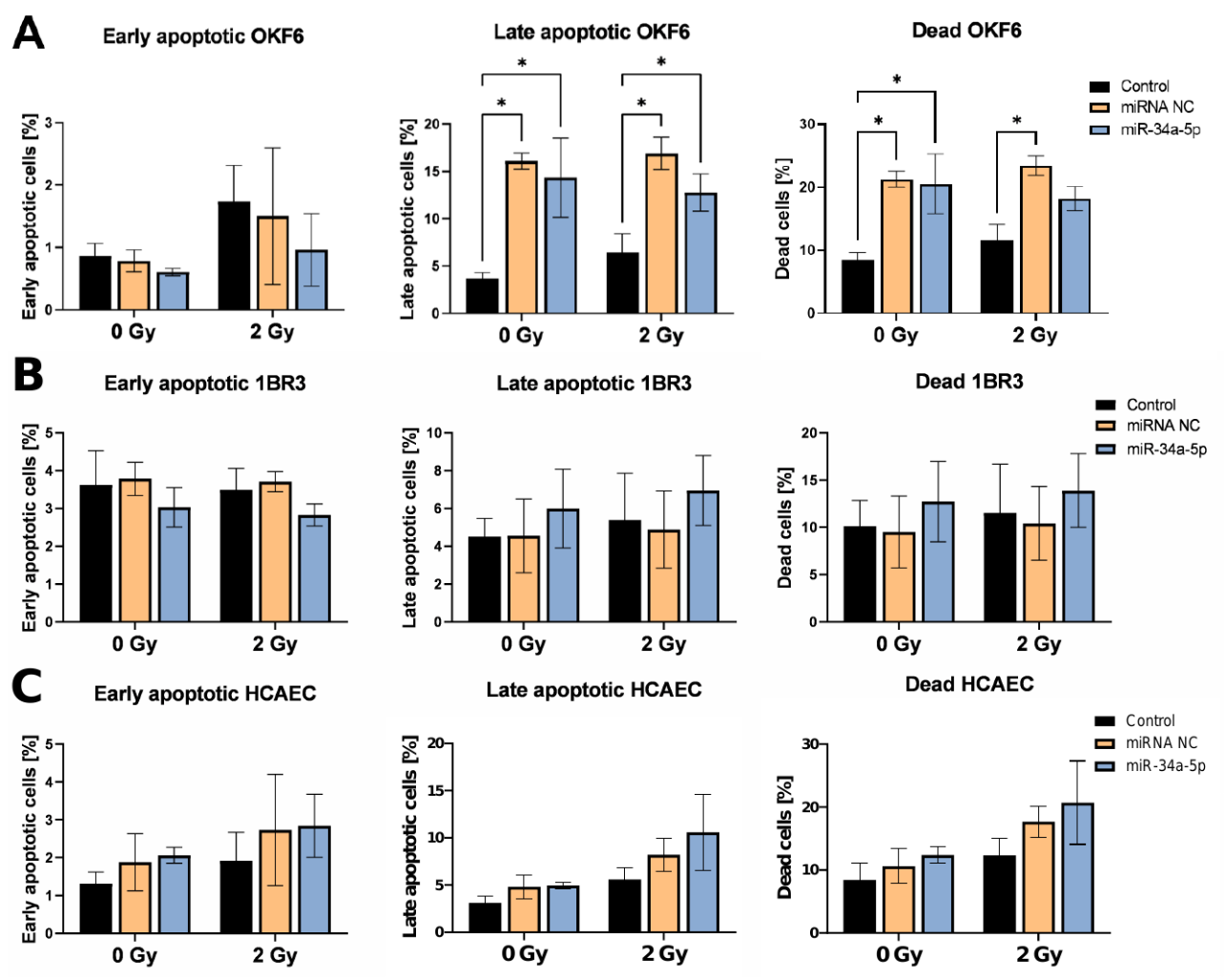


**Supplementary Fig. 7: Apoptosis in miR‑34a‑5p transfected recipient cells.** Percentage of apoptotic cells measured 72 h after miR‑34a‑5p transfection of OKF6, 1BR3 and HCAECs by staining phosphatidylserine with Annexin V. Cells were transfected with 10 nM of a scrambled miRNA negative control or 10 nM miR‑34a‑5p and irradiated with 0 or 2 Gy after 1 h incubation. Mean values ± SD of three biological replicates are shown. For statistical analysis, 2‑way ANOVA and multiple t-tests with Benjamini-Hochberg correction was performed.

*
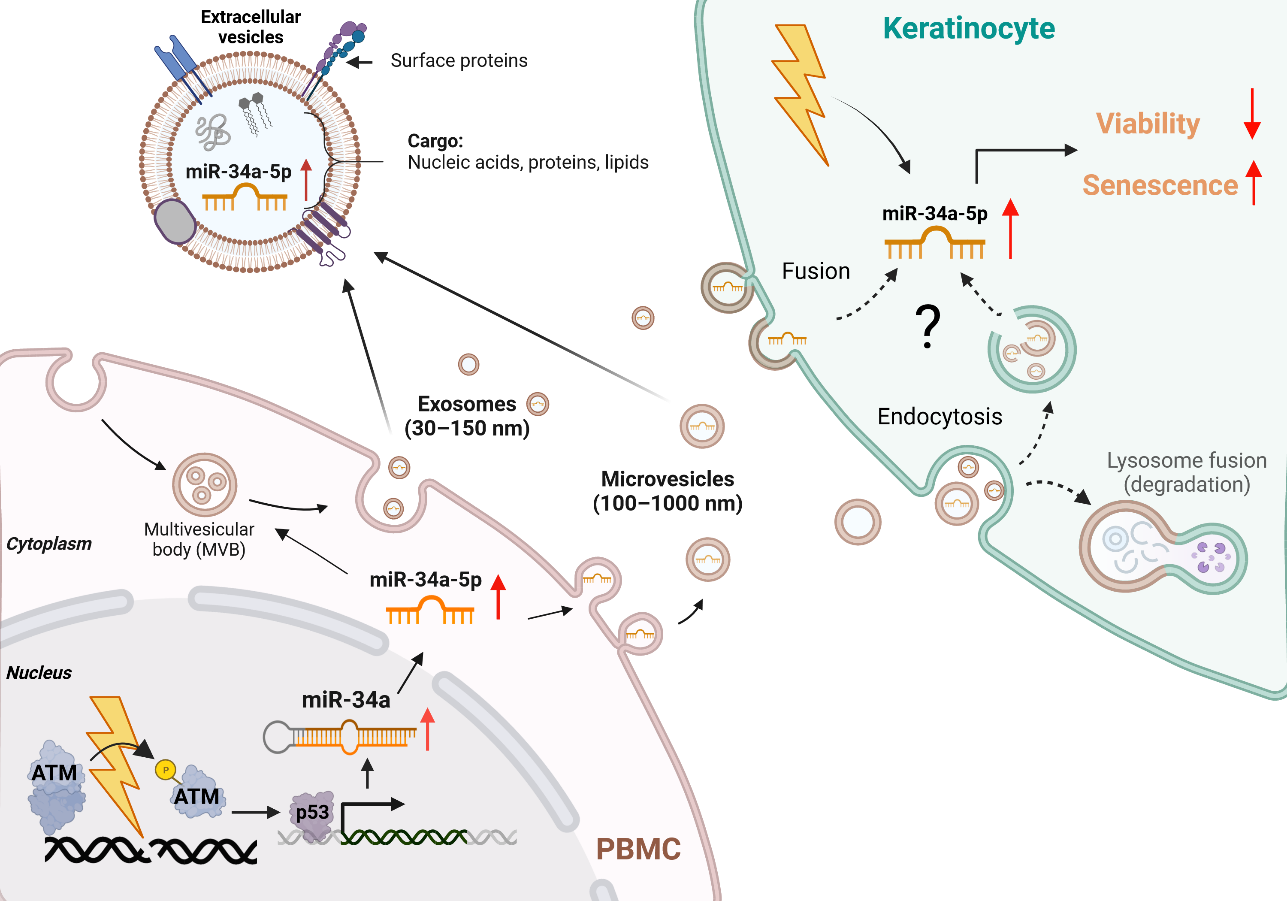
*

***Supplementary Fig. 8:*** *Suggested model for the EV-based communication between PBMCs and keratinocytes with focus on miR-34a-5p (for details see discussion 5.3)*
