# Supplementary material for: Ionizing radiation effects on blood-derived extracellular vesicles: insights into miR-34a-5p-mediated cellular responses and biomarker potential
Source: Cell Commun Signal. 2024 Oct 2;22:471. doi: 10.1186/s12964-024-01845-x (PMC11446100; doi:10.1186/s12964-024-01845-x)
Supplement: Supplementary file 2 — Additional file 2. Raw images of western blots. [file 12964_2024_1845_MOESM2_ESM.docx]

Additional file 2: raw image files of western blots


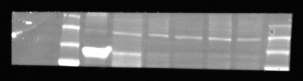

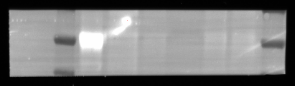


Figure 1D: Calnexin

Figure 1D: Tubulin

Figure 1D: Tubulin

Figure 1D: CD81


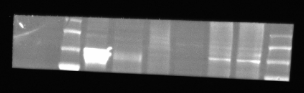


Figure 1D: Alix


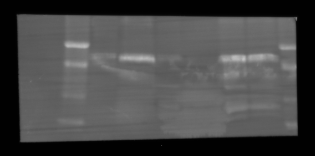


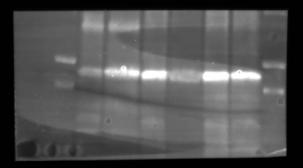


Figure 1D: CD9


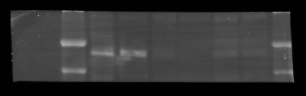


Figure 1D: TSG101


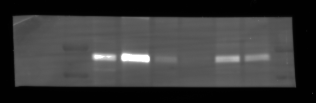


Figure 1D: Flotilin


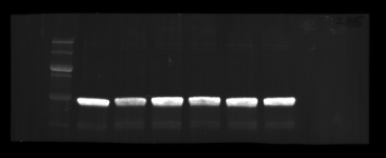


Figure 4A: Steap3


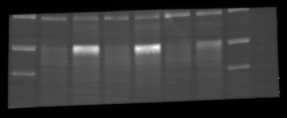


Figure 4A: p53


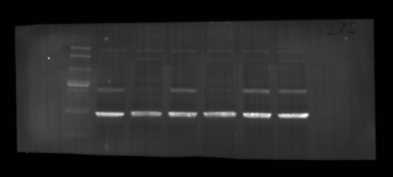


Supp. Figure 4A: Steap3
